# Emergence of chaos in a compartmentalized catalytic reaction nanosystem

**DOI:** 10.1038/s41467-023-36434-y

**Published:** 2023-02-10

**Authors:** Maximilian Raab, Johannes Zeininger, Yuri Suchorski, Keita Tokuda, Günther Rupprechter

**Affiliations:** 1 Institute of Materials Chemistry, TU Wien, Getreidemarkt 9, 1060 Vienna, Austria; 2 Department of Computer Science, University of Tsukuba, 1-1-1 Tennodai, Tsukuba, Ibaraki 305-8577 Japan

**Keywords:** Heterogeneous catalysis, Chemical physics, Catalytic mechanisms, Nanoparticles

## Abstract

In compartmentalized systems, chemical reactions may proceed in differing ways even in adjacent compartments. In compartmentalized nanosystems, the reaction behaviour may deviate from that observed on the macro- or mesoscale. In situ studies of processes in such nanosystems meet severe experimental challenges, often leaving the field to theoretical simulations. Here, a rhodium nanocrystal surface consisting of different nm-sized nanofacets is used as a model of a compartmentalized reaction nanosystem. Using field emission microscopy, different reaction modes are observed, including a transition to spatio-temporal chaos. The transitions between different modes are caused by variations of the hydrogen pressure modifying the strength of diffusive coupling between individual nanofacets. Microkinetic simulations, performed for a network of 52 coupled oscillators, reveal the origins of the different reaction modes. Since diffusive coupling is characteristic for many living and non-living compartmentalized systems, the current findings may be relevant for a wide class of reaction systems.

## Abstract (web summary)

In situ studies of the spatio-temporal behavior of individual well-defined nanosized compartments are paramount in heterogeneous catalysis. Here, a transition from oscillating to chaotic behaviour was observed in catalytic hydrogen oxidation on a rhodium nanocrystal serving as a model of a single catalytic particle.

## Introduction

Compartmentalisation, i.e., the formation of cells as dynamic compartment systems, which are separated from the environment by boundaries that control the exchange of energy and matter, is one of the fundamental principles of life^1–3^. Due to compartmentalisation, chemical reactions in biological systems can proceed in adjacent compartments in parallel, but in a spatially differing way^4–7^, depending on the degree of the intercompartment communication, e.g., via active transport mechanisms or diffusion^8,9^. Examples of compartmentalisation can also be found in the non-living nature, e.g., in oscillating reactions in microemulsions, droplet arrays and gels, which mimic important hallmarks of biological behaviour, such as communication and motion^10^.

In catalysis, compartmentalisation may even allow incompatible catalytic transformations to occur in close proximity at the same time, e.g., by using a core-shell structure^11^ of two well-defined metal-oxide interfaces within one particle^12^ or two-phase aquatic systems^13^.

A suitable way to study compartmentalisation in catalytic reactions is to investigate their spatio-temporal behaviour, such as kinetic transitions^14–16^, self-sustained oscillations^17–19^, multistability^20^ and Turing patterns^21^, resulting in the formation of ordered spatio-temporal structures, which somewhat mimics the metabolism^10^. All these phenomena take place in two dimensional systems, simplifying the full atomistic understanding of the ongoing processes, including corresponding micro-kinetic modelling^19,22,23^.

Until now, such spatio-temporal phenomena have been mostly studied on macroscopic single crystal surfaces, i.e., in systems that are spatially unconfined from a microscopic point of view^19^. Already a mesoscopic compartmentalisation of the reaction system by using a polycrystalline metal foil consisting of individual µm-sized crystalline grains provided novel features: multifrequential oscillations^24^, frequency transforming by grain boundaries^25^ and simultaneously existing multi-states^20^.

Since nanoparticles are the main working horses in catalysis, corresponding model systems, which allow in situ studies of the spatio-temporal behaviour of individual well-defined nanosized compartments are required. The apex of a catalytically active metal tip appears to be a suitable model: it possesses the main property of a nanoparticle, namely the coexistence of differently oriented nanofacets and also allows an atomic scale characterisation and real time monitoring of the reaction process on individual nanofacets^16,26,27^. The nanofacets, separated from each other only by atomic ridges, represent individual nanosized reaction compartments, the size and orientation of which can be controlled by appropriate sample preparation. In combination with dedicated spatially-resolving techniques, this provides a wide playground for studies of compartmentalisation in catalytic nanosystems.

However, all observable reaction instabilities, such as kinetic transitions^28–31^ or self-sustaining oscillations^32–36^ were observed to occur in a synchronised way over the entire particle surface, so that they were not treated as compartmentalised systems. Only the recent detection of multifrequential oscillations in the H_2_ oxidation reaction, where different facets of 10–100 nm diameter of a µm-sized curved Rh crystal behaved independently, disproved this wide-spread opinion^37^. In this system, additional compartmentalisation-related effects were observed, including limited interfacet coupling, entrainment and frequency-locking^37^.

An important consequence of the non-linearity of chemical reactions is the possibility of chaotic behaviour, as considered theoretically and observed experimentally on Pt single crystal surfaces in the CO oxidation reaction^38–40^. In principle, this behaviour can also be expected in a compartmentalised reaction system^41^, since interactions between confined oscillators can lead to the spontaneous breakdown of synchronisation, as proposed by Winfree^42^ and modelled by Kuramoto^43,44^. Such synchronisation breakdowns can be induced by a reduction of the coupling strength, as predicted in model calculations^45,46^. Experimentally, Gollub et al. demonstrated the possibility of such a breakdown, resulting in chaos for a system of coupled diodes^47^. Kuramoto´s theory was finally verified by Kiss and co-workers using a population of coupled chemical oscillators^48^, i.e., on the macroscale. A transition from synchronous behaviour to chaos in a nanosized system was, however, not yet observed.

In the present study we demonstrate that compartmentalised reaction behaviour can be observed not only on a µm-sized curved crystal, but also on the nanofacets of a significantly smaller Rh nanotip. We applied field ion microscopy (FIM) with atomic resolution to characterise the shape, size and the atomic structure of the individual nanofacets serving as reaction compartments, subsequently using in situ field emission microscopy (FEM) to image the ongoing H_2_ oxidation with ~2 nm resolution. Monofrequential and multifrequential self-sustained kinetic oscillations were observed when varying the temperature and hydrogen pressure and, for a particular region of the *p*_H2_/T space, chaotic behaviour was evoked by the breakdown of synchronisation between compartments. The experimental studies are complemented by microkinetic modelling of hydrogen oxidation on Rh nanofacet compartments acting as coupled individual oscillators.

## Results

### An ellipsoid-like rhodium nanocrystal as a model system

**Fig. 1:**
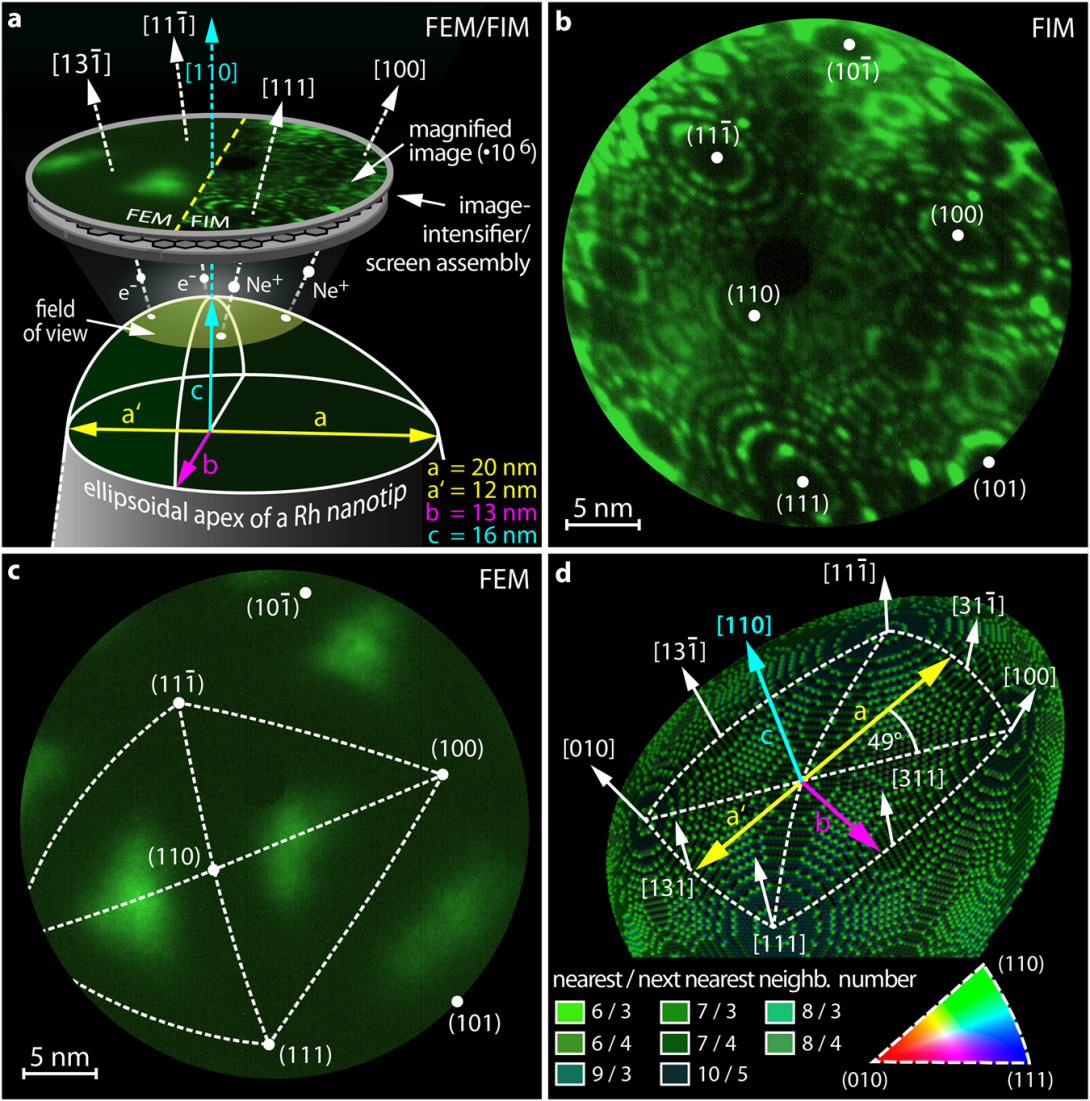
Apex of an ellipsoidal Rh nanocrystal. **a** Schematics of the experimental setup and the sample geometry: in FEM and FIM, field emitted electrons and ions, respectively, form a point projection image of the sample surface. The apex of the [110]-oriented Rh nanocrystal has axis lengths of a, a′, b and c. **b** FIM image of the Rh nanocrystal, obtained at *T* = 77 K using Ne^+^ ions. **c** the same field of view, but in the FEM mode, with the same crystallographic markings as in **b**. **d** Ball model of the nanocrystal apex with crystallographic net overlay, where each of the four triangles forming the net corresponds to the inverse pole figure (bottom right). To illustrate the local atomic corrugation, the individual atoms are colour-coded according to their nearest and next-nearest neighbour numbers.

A Rh nanocrystal was intentionally formed as a slightly asymmetrical ellipsoid (Fig. 1a), to achieve divergence in the natural oscillation frequencies of similar compartments (i.e., nanofacets). Such divergence, i.e., a broader distribution of natural frequencies of individual oscillators, is known to be generally advantageous for chaotic behaviour, at least in areas other than chemical reactions^45,46^.

FIM visualises the surface using field-ionised noble gas atoms (Ne in the present case) for the formation of a magnified point projection image (Fig. 1a) and provides, due to atomic resolution (Fig. 1b), the positions of protruding surface atoms^49^. FEM utilises field-emitted electrons to map the local work function distribution (Fig. 1c)^50^. The FIM/FEM images of the clean sample allow determining the apex shape, dimensions, and crystallographic orientations of the nanofacets (see Supplementary Note 1 and Supplementary Fig. 1).

Based on these parameters, a 3D atomic ball model was calculated, exactly indicating the position and coordination number of all surface atoms (Fig. 1d). The slightly asymmetric ellipsoid-like apex surface consists of two parts with different a axes (Fig. 1a, d). Consequently, the four triangles, each corresponding to the inverse pole figure (IPF) and forming the surface crystallographic net (Fig. 1d), obey different atomic blueprints. Ultimately, the resulting apex surface forms a collection of crystallographically different nanofacets, i.e., a nano-library of surface structures^51^. In contrast to symmetrical nanotips that are commonly used in FIM/FEM studies, in the present case the multiple facets of the same orientation, e.g., four 〈311〉-oriented facets, have different sizes and therefore variations in their step and kink configuration, a divergence that will later prove to be essential for the observed effects.

### Spatio-temporal instabilities on the nm-scale

FEM allows in situ real-time visualisation of catalytic reactions, as the image contrast results from the local work function which allows direct identification of different adsorbate species^16^. In catalytic hydrogen oxidation, the oxygen-covered, catalytically inactive surface has a dark contrast, while the active surface with a low hydrogen coverage appears bright. The hydrogen oxidation on Rh follows the Langmuir-Hinshelwood mechanism: both hydrogen and oxygen molecules dissociatively adsorb on the surface, and then react to water which immediately desorbs at the present temperatures of 410–480 K^52^. Apart from the catalytically inactive (oxygen covered) and catalytically active (hydrogen covered) steady states, at specific external conditions (constant temperature and reactant partial pressures) hydrogen oxidation on rhodium may exhibit self-sustaining oscillations between the catalytically active and inactive reaction states^20,24,25,37,53,54^. As in other catalytic reactions, such oscillations and kinetic transitions usually occur synchronised in heterogeneous nanosized systems^23,33,54^, due to coupling via surface diffusion^27,33^ or via gas phase^55^. Recently, it was detected that the disruption of diffusion due to grain boundaries (in mesoscale systems) or protruding atomic rows (in nanoscale systems) causes a collapse of spatial coupling, generating multifrequential oscillations, where neighbouring µm- or even nm-sized regions of the same sample oscillate with their own frequencies^20,24,25,37^.

Besides the periodic modes of oscillations, under certain conditions surface reactions can exhibit chaotic behaviour, with non-periodic switches between the active and inactive state and absence of spatial correlation between local reaction states^38,39^. The smallest changes in the reaction conditions, e.g., temperature, reactant partial pressures, lead to a remarkable different spatio-temporal evolution of the aperiodic reaction process, i.e., to deterministic chaos^56^. In theoretical modelling, such chaotic behaviour can be triggered, e.g., by a reduction in coupling between individual oscillators^45,46^. Experimentally, deterministic chaotic behaviour has been observed on the mesoscale in CO oxidation on Pt single crystals under UHV^38,39^ and on porous Pt/Al_2_O_3_ under atmospheric pressure^57^. On the nanoscale, however, due to strong diffusion coupling and a diffusion length larger than the entire sample, the occurrence of such behaviour seemed to be unobservable.

**Fig. 2:**
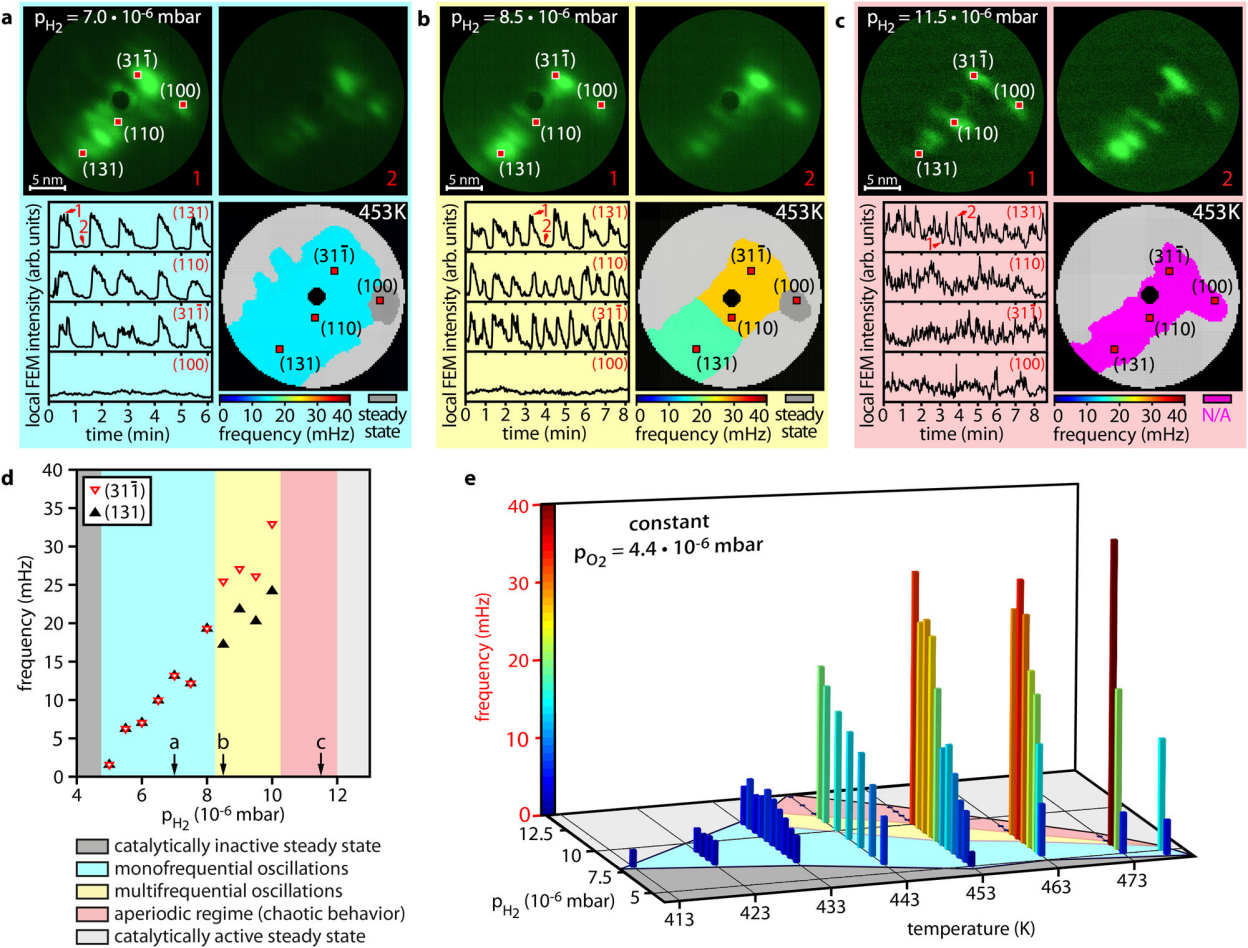
Different modes in catalytic H_2_ oxidation on a Rh nanocrystal at constant *p*_O2_ = 4.4 × 10^−6^ mbar. **a** Monofrequential oscillations at *p*_H2_ = 7.0 × 10^−6^ mbar and 453 K. (Top) in situ FEM video frames (27 by 27 nm). (Bottom left) Local FEM intensity curves measured within ROIs (0.9 by 0.9 nm) placed in (110), (100), (131) and $(31\bar{1})$ regions. The time points corresponding to video frames 1 and 2 are marked in red. (Bottom right) Colour-coded frequency map. **b** Multifrequential oscillations at *p*_H2_ = 8.5 × 10^−6^ mbar and 453 K. **c** Aperiodic behaviour at *p*_H2_ = 11.5 × 10^−6^ mbar and 453 K. **d** Pressure dependence of the oscillation frequencies within the (131) and $(31\bar{1})$ ROIs at 453 K. Colour codes separate regions of different modes. Black arrows indicate the *p*_H2_-values corresponding to **a**–**c**, respectively. **e** Diagram illustrating the existence ranges of the observed reaction modes. Different states are colour-coded the same way as in **d**. The height and colour of the bars represent the oscillation frequency.

In this study, the FIM/FEM chamber was operated as a catalytic flow-reactor at constant oxygen pressure of 4.4 × 10^−6^ mbar and hydrogen pressures ranging from 4.0 × 10^−6^ to 1.5 × 10^−5^ mbar. First, measurements were performed at *T* = 453 K, varying only *p*_H2_. At *p*_H2_ = 7.0 × 10^−6^ mbar, self-sustaining oscillations were observed, synchronised across almost the whole sample apex surface (Fig. 2a and Supplementary Movie 1). The FEM intensity extracted from regions of interests (ROIs) placed in the (110), (131) and $(31\bar{1})$ regions show periodic oscillations with the same frequency of 13.5 mHz, while the reaction does not oscillate on the (100) facet (Fig. 2a bottom left and frequency map bottom right). The frequency map was constructed by analysing the local FEM intensity in the full field of view, details on the frequency map construction are given in Supplementary Note 2 and Supplementary Fig. 2. The analysis algorithm first distinguishes between oscillating and aperiodic behaviour and determines the local oscillation frequency via Fourier analysis, provided that oscillating behaviour is present. The synchronised monofrequential oscillations shown in Fig. 2a are similar to those previously observed on symmetrical nanocrystals^23,27,54^.

When increasing *p*_H2_ to 8.5 × 10^−6^ mbar, the global synchronisation breaks down and local multifrequential oscillations can be observed (Fig. 2b and Supplementary Movie 2). The reason lies in the atomic grooves on the {110} and {311} facets resulting from the oxygen induced and thermally activated missing-row reconstruction, present above the reconstruction temperature of 433 K^58,59^. These atomic grooves create a barrier whose anisotropic diffusion-disturbing effect is not sufficient to desynchronise oscillations at *p*_H2_ = 7.0  × 10^−6^ mbar, leading solely to a stop-and-go like propagation of reaction fronts. However, at 8.5 × 10^−6^ mbar, they strongly reduce the spatial synchronisation between the two regions (see Supplementary Note 5 for details). The desynchronization results in the frequency map consisting of two internally correlated regions with different frequencies, separated by the diffusion barrier (Fig. 2b) and a small steady state region with characteristic microscopic fluctuations^60^. The present observation of multifrequential oscillations is astonishing, because the size of the nanocrystal investigated herein is an order of magnitude smaller than the tip used in our first observation of this phenomenon^37^. On the correspondingly small facets, the missing-row reconstruction affects only a few atomic rows, i.e., the diffusion barrier is narrow and the diffusion coupling is thus expected to be more effective, providing global synchronisation, as reported in previous publications^23,27,33,36,54^.

### Emergence of spatio-temporal chaos

At *p*_H2_ = 11.5 × 10^−6^ mbar, another different reaction behaviour occurs (Fig. 2c and Supplementary Movie 3): The local FEM intensity starts to make aperiodic and fast-paced jumps, indicating sudden local changes of catalytic activity from the inactive to the active state and vice versa. This effect is present across the entire sample surface as illustrated by four intensity curves in Fig. 2c. While the observed behaviour is fundamentally the same in all four ROIs, they seem to act independently from one another, leading to different arrangements of facets to be active at each point in time, which is exemplarily shown in the two FEM video frames in the top panel of Fig. 2c. The phenomenon is aperiodic, i.e., a local frequency is not assignable for any facet.

The above different reaction modes (Fig. 2a–c) can be assigned to specific hydrogen pressure ranges: at 453 K, increasing hydrogen pressure causes a transition from the inactive steady state to monofrequential oscillations at 4.9 × 10^−6^ mbar and above 8.0 × 10^−6^ mbar a multifrequential mode is initiated, which changes to aperiodic behaviour above 1.0 × 10^−5^ mbar. Eventually a transition to the active steady state occurs at 1.2 × 10^−5^ mbar (Fig. 2d).

At other temperatures, the pressure ranges corresponding to particular modes change, but still form a specific parameter region for each mode, as summarised in a diagram (Fig. 2e). Below 433 K, only monofrequential oscillations were observed, which may be traced back to the absence of thermally activated reconstructions causing the collapse of diffusional coupling^37^, which do occur around 433 K^58,59^. Above 433 K, three different modes are observed, and a temperature increase narrows the parameter regions for each of three observed reaction modes and shifts them towards lower hydrogen pressure (Fig. 2e). This temperature effect results from the temperature dependent adsorption/desorption equilibrium for hydrogen and oxygen. Due to a faster formation/depletion of subsurface oxygen at higher temperatures, the average and maximum oscillation frequencies increase with temperature. While the mono- and multifrequential modes are well-determined, the characterisation of the third, aperiodic mode needs more efforts.

The present aperiodicity raises the question about the possibility of a chaotic state. However, the verification of chaotic reaction behaviour in nanosystems is a great challenge due to the drift of reaction parameters limiting the monitoring duration and reaction-induced fluctuations superimposing the reaction process^60^. Such fluctuations occur in the bistable mode of the reaction and exhibit comparably much faster rate than self-sustained oscillations. Fluctuations are confined to individual nanofacets and are not synchronised between different, even adjacent, regions^61,62^. Additionally, surface density fluctuations caused by the diffusion of mobile adsorbates take place in any mode of the reaction. Such fluctuations have monomodal Gaussian-like amplitude distributions, negligible spatial correlation and can be well-observed in the steady states, where reaction-induced bistable fluctuations do not take place^60–63^. These distinguishing features of diffusion- and reaction-induced fluctuation allow a distinction between fluctuations and chaotic behaviour. The identification of chaos, in turn, is somewhat more difficult, since the number of chaos indicators suitable for experimental data is limited. Nevertheless, the analysis of the autocorrelation function and the Fourier spectra can indicate if chaotic states occur (for details see Supplementary Note 3). Indeed, the autocorrelation function calculated for the local timeseries shown in Fig. 2c exhibits strong dampening and an absence of periodic peaks and Fourier analysis of the same timeseries via fast-Fourier transformation yields broad low-frequency peaks disappearing in a broadband continuous spectrum (for details see Supplementary Note 3 and Supplementary Fig. 3). Both features indicate the presence of a chaotic state in the present experimental observations. Additional indications for suspected chaotic behaviour can be distilled from the decay of spatial correlation of ongoing processes^64^, as is, e.g., used to identify chaotic motion in fluid mechanics^65^.

### Spatial correlation reflects different reaction modes

**Fig. 3:**
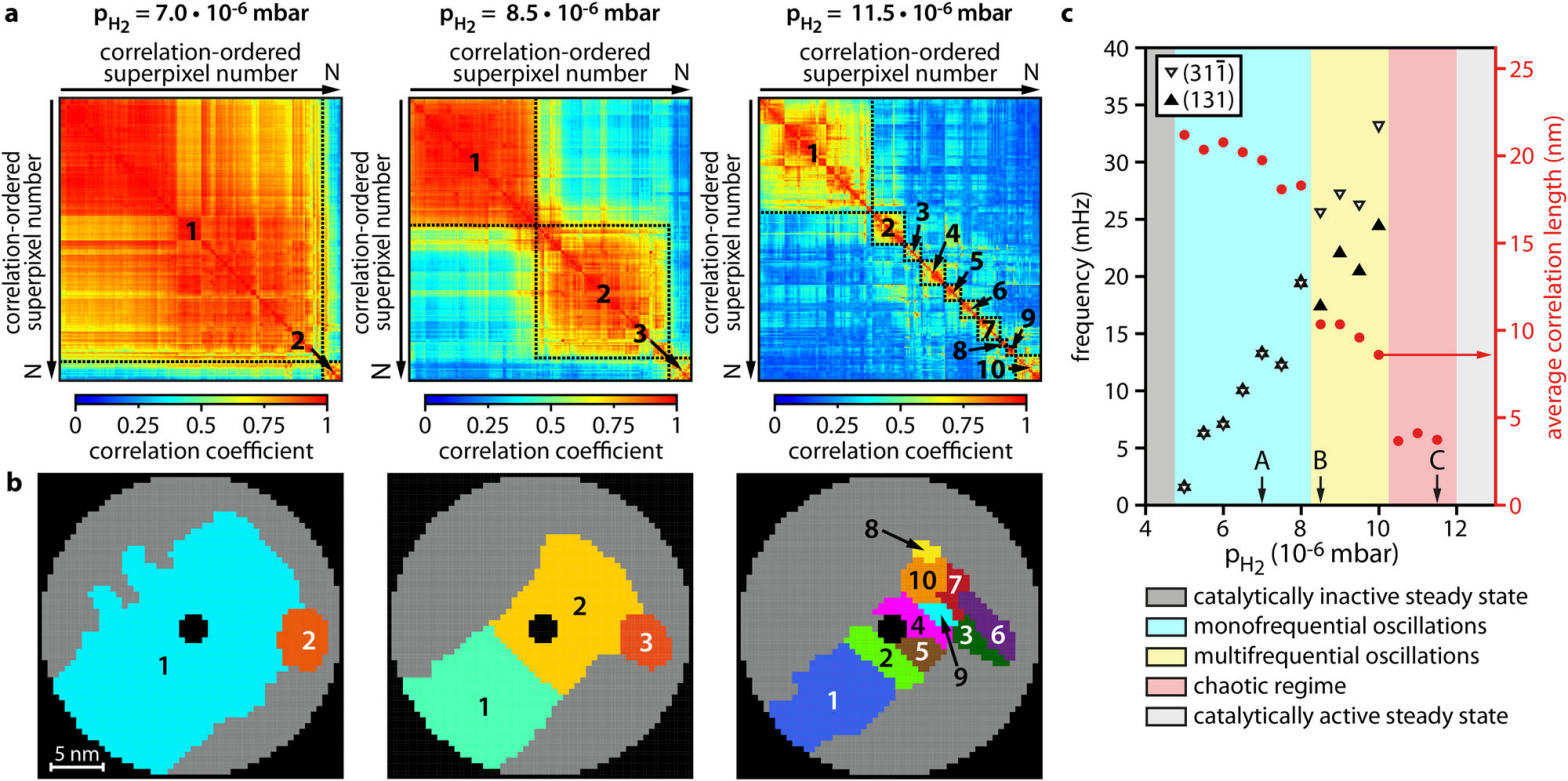
Spatial correlations at constant *p*_O2_ = 4.4 × 10^−6^ mbar and 453 K. **a** correlation coefficient matrix summarising the correlation between experimental timeseries of each individual super pixel (8 × 8 pixels), the colour represents the value of the correlation coefficient. (Left) *p*_H2_ = 7.0 × 10^−6^ mbar, (middle) *p*_H2_ = 8.5 × 10^−6^ mbar, (right) *p*_H2_ = 11.5 × 10^−6^ mbar. The superpixels are ordered according to spatial clustering, grouping surface regions with highly correlated behaviour, with the borders between the clusters marked; **b** Correlation map displaying the numbered and colour marked individual clusters. **c** Average correlation length values (right vertical scale) for different hydrogen pressures are plotted as red circles together with the oscillation frequencies from Fig. 2d (left vertical scale). Black marked arrows indicate the *p*_H2_-values corresponding to **a**.

Therefore, spatial correlation coefficients – defined as the maximum values of the cross-correlation functions (Supplementary Note 4) – were calculated for the experimental timeseries of the three different parameter sets corresponding to Fig. 2. From this, spatial correlation matrices were constructed (see Supplementary Note 4 and Supplementary Fig. 4a–d) and portrayed in a way that maximises the correlation between adjacent matrix rows, whereby their order is optimised for each matrix separately (Fig. 3a). The matrices in Fig. 3a demonstrate how the degree of spatial correlation weakens with increasing H_2_ partial pressure. The weakening, however, does not occur uniformly, but the network of nanofacets undergoes a splitting from a strong global correlation to several internally correlated clusters. This is illustrated by a correlation map (Fig. 3b), which can be constructed using an agglomerative hierarchical clustering algorithm. The algorithm is based on the construction of a dendrogram from the individual correlation coefficients (Fig. 3a, see Supplementary Note 4 and Supplementary Fig. 4e, f). This allows us to trace the spatial correlation over the entire experimentally investigated *p*_H2_ range, including the aperiodic mode.

From the evaluation of the spatial extension of correlation for each *p*_H2_, an average correlation length was calculated. Figure 3c shows the behaviour of the correlation length with increasing *p*_H2_, whereby the two jumps in the curve exactly coincide with the *p*_H2_ pressures at which the transitions between the different modes occur, i.e. the measured correlation length reflects the reaction mode. A collapse of spatial coupling and a consequent disaggregation of local oscillators is characteristic for collective chaotic behaviour: a transition from an oscillation mode to a chaotic one is usually accompanied by a sudden decrease of the spatial correlation^64,65^.

As mentioned above, the present aperiodic oscillations showing chaotic features can be clearly distinguished from reaction- and diffusion induced fluctuations. An example of the analysis of fluctuations occurring in the active state and exhibiting negligible spatial correlation is described in Supplementary Note 5. This property is significantly different from the spatial correlation in the aperiodic reaction mode, where regions of substantial correlation are still present (cf. Fig. 3a and Supplementary Fig. 5).

### Coupled oscillators: micro-kinetic modelling

**Fig. 4:**
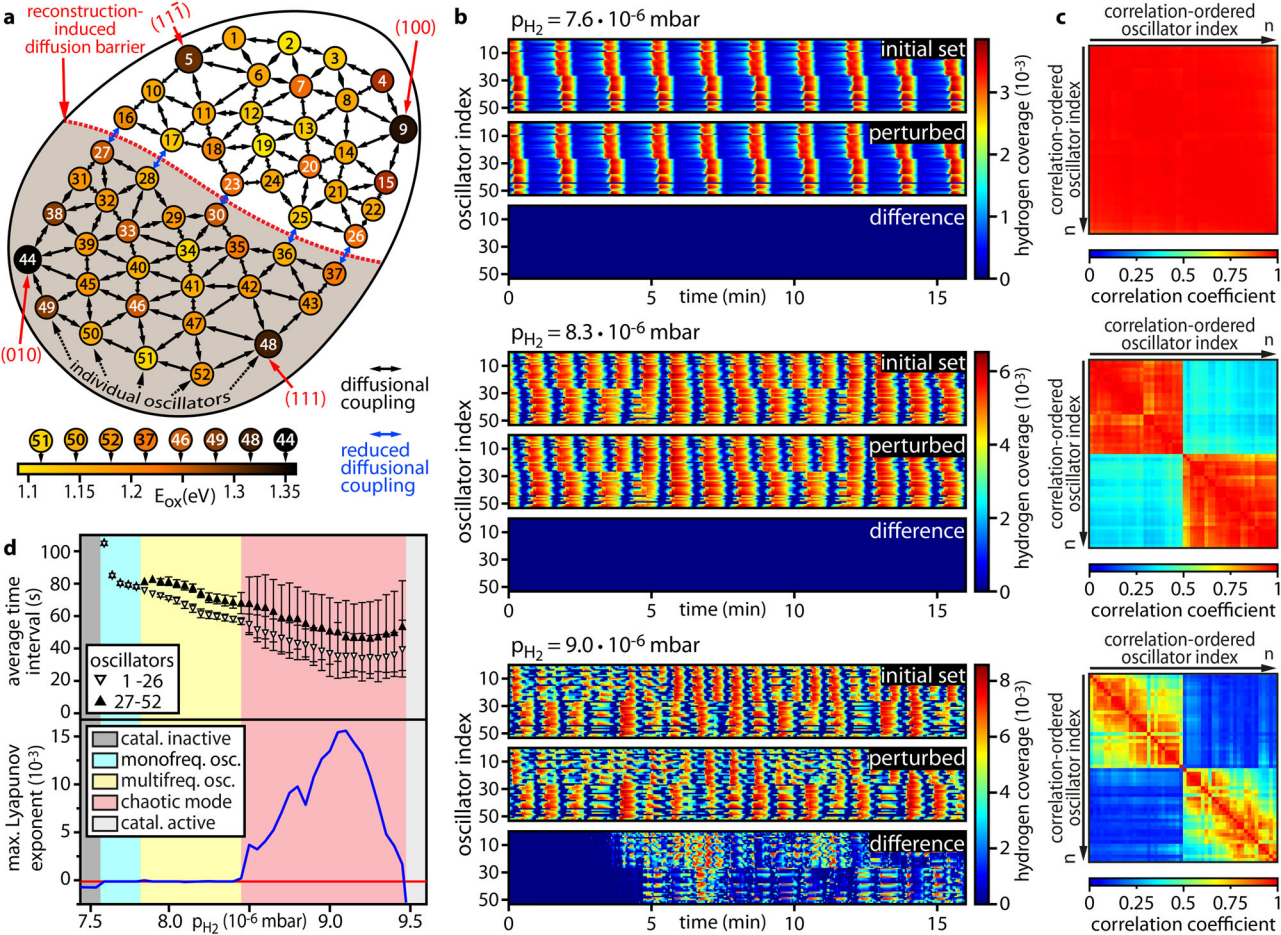
Micro-kinetic modelling of the reaction on a 2D-oscillator grid. **a** Layout of the oscillator grid based on the surface crystallography shown in Fig. 1. Numbered individual oscillators are coloured according to their activation energy for subsurface oxygen formation. Oscillators are coupled via hydrogen diffusion, indicated by double arrows. The red dotted line represents the diffusion barrier. **b** Calculated timeseries of the local hydrogen coverages for each oscillator at 453 K and constant *p*_O2_ = 4.4 × 10^−6^ mbar and *p*_H2_ = 7.6 × 10^−6^ mbar (top); *p*_H2_ = 8.3 × 10^−6^ mbar (middle)¸ *p*_H2_ = 9.0 × 10^−6^ mbar (bottom). The lower strips in each panel show the difference between the timeseries calculated for the set of initial conditions (upper strip in the panel) and timeseries calculated for slightly perturbed initial conditions (middle strip). **c** correlation coefficient matrix representing the correlation between hydrogen coverages of each individual model oscillator at the respective *p*_H2_, ordered to maximise the correlation between adjacent matrix rows (colour represents the correlation coefficient). **d** Calculated bifurcation diagram (top); largest Lyapunov exponent (bottom, blue line) for *T* = 453 K, constant *p*_O2_ = 4.4 × 10^−6^ mbar and different *p*_H2_. Colour codes separate regions of different modes. The bars show mean ± standard deviation.

To rationalise the present experimental observations and the above indications for chaotic behaviour, we performed microkinetic modelling for the system of coupled oscillators, adapting the reaction model previously used for simulating single, uniform oscillators^23^ (see Supplementary Note 6). To account for the present complex system, we extended the model by translating the arrangement of different crystallographic nanofacets on the sample surface (Fig. 1d) into a network of 52 oscillators, coupled by surface diffusion of hydrogen (Fig. 4a).

The microkinetic model simulations are based on the Langmuir-Hinshelwood mechanism that is well-established for catalytic hydrogen oxidation on Rh. This model concept was originally introduced by McEwen et al.^23^ to simulate field-modified oscillations, observed by FIM in the presence of a high electric field of  >10 V/nm^23,36,54^. The present studies were performed by FEM, where the applied field is much lower (<5 V/nm) and of opposite direction. Due to the field direction away from the sample surface, the field strength that can be applied in a FEM is limited by the onset of avalanche-like field emission (see Supplementary Note 7) to values at which field-induced changes of electron density near the surface and thus field-induced modifications of adsorption energetics do not yet arise^30,66,67^. The absence of field effects in the current observations was directly proven by pulsed field experiments, using a pulsed high-voltage supply with varying duty pulses, in analogy with earlier experiments on CO oxidation^67^. No differences between results obtained using both modes of FEM imaging were observed, neither for kinetic transitions nor for oscillations (see Supplementary Note 7 for details). Since field-effects can therefore be neglected in the present case, the field-dependent terms were omitted in the current modelling.

Periodic formation and depletion of subsurface oxygen acts as feedback mechanism governing the oscillations (see Supplementary Next 6 and Supplementary Fig. 6). The rate of subsurface oxygen formation and thus the natural oscillation frequency is determined by the activation energy for the formation of subsurface oxygen E_ox_^20,24,25,37^. The rate of surface oxidation has been shown to be related to the local atomic surface roughness, i.e., the crystallographic orientation of the Rh surface^68^. Accordingly, an E_ox_ value was assigned to each oscillator of the model network, ranging from 1.09 to 1.36 eV (Fig. 4a), depending on the crystallographic facet it represents. The coupling between individual oscillators is incorporated into the model by addition of a diffusion term that describes the interfacet exchange of hydrogen (double arrows in Fig. 4a). The existence of the reconstruction-induced barriers was simulated by locally reduced diffusion (Fig. 4a, blue arrows). Oxygen diffusion, which is slower than that of hydrogen by at least three orders of magnitude at present conditions^69^, could be neglected: Additional calculations taking the oxygen diffusion into consideration, analogous to Makeev and Imbihl^69^, did not affect the agreement between experiment and modelling.

The calculation results at constant *p*_O2_ = 4.4 × 10^−6^ mbar and 453 K are presented in Fig. 4b and Supplementary Movie 4. With increasing hydrogen pressure, the simulated behaviour changes and passes through three different modes. For low hydrogen pressure, calculations predict monofrequential oscillations for all oscillators (top panel of Fig. 4b) in full agreement with our experimental observations.

At *p*_H2_ = 8.3 × 10^−6^ mbar, the calculations result in multifrequential oscillations (Supplementary Movie 5), where the upper and the lower regions (Fig. 4a) oscillate independently with differing frequencies (middle panel of Fig. 4b), which is again in line with our experimental findings. At increased hydrogen pressures, the oscillators within both regions no longer match each other, not even with their immediate neighbours (bottom panel of Fig. 4b and Supplementary Movie 6). Evidently, switches between the active and inactive state still occur, however, they are no longer periodic or synchronised. To find indications for chaotic behaviour, we repeated the simulations, including small perturbations in the initial surface coverages (for details see Supplementary Note 6). For the first two modes, the resulting influence on the temporal evolution of the surface coverages is negligible, as illustrated by the difference stripes in the top and middle panels of Fig. 4b. For the third, aperiodic mode, drastic changes in the behaviour occur (difference stripe in the bottom panel in Fig. 4b) indicating the existence of spatio-temporal chaos.

Analogous to the experimental data, spatial correlation matrices were calculated for each pair of 52 oscillators for the three different parameter sets corresponding to Fig. 4b and are shown in Fig. 4c. The matrices show a similar type of pattern as in the experiment, namely a splitting at rising hydrogen pressure from a strong global correlation to few internally correlated patches (Fig. 4c), reproducing the experimentally observed clustering. A more pronounced drop in the experimentally determined correlation of the regions along the diffusion barrier, shown in the bottom panel of Fig. 4c, is caused by the non-ideality of the experimental conditions containing apparative noise and diffusion-caused fluctuations^70^.

The bifurcation diagram in the top panel in Fig. 4d, where the average time between two consecutive kinetic transitions to the active state serves as measure of periodicity, summarises the calculations.

To prove the existence of chaos, Lyapunov exponents were calculated from the simulated timeseries via the standard numerical method (bottom panel in Fig. 4d, for details see Supplementary Note 7). Lyapunov exponents characterise the separation rate of trajectories that are infinitesimally close to each other. For periodic systems, exhibiting converging or stable trajectories, Lyapunov exponents show negative or zero values, while for chaotic systems, where these trajectories diverge, the maximum Lyapunov exponent exhibits a positive value^71^. This is the case for the simulated timeseries at *p*_H2_ = 9.0 × 10^−6^ mbar, where small deviations of the initial conditions lead to increasing divergence of the H-coverage (lowest panel in Fig. 4b), which corresponds to positive Lyapunov exponents (Fig. 4d), thus providing an unambiguous sign for chaotic behaviour.

Since the chaotic state established in the model calculations is characterised by a specific correlation coefficient matrix consisting of small highly correlated regions (oscillator clusters, Fig. 4c), the appearance of this feature in the experiment (Fig. 3a) may serve as additional evidence of chaotic behaviour (for details see Supplementary Note 6).

The fact that increasing the hydrogen pressure weakens the spatial synchronisation, leading to multifrequential oscillations and then to a chaotic state, might seem counterintuitive at first, but can be rationalised as follows: for each oscillator (nanofacet), there are two sources of hydrogen, namely hydrogen adsorption from the gas phase and surface diffusion from neighbouring facets. Without diffusion, the natural frequency of each nanofacet is governed by its local crystallography and the adsorption rate of hydrogen. However, in reality, each nanofacet in the active state, i.e., with a considerable hydrogen coverage, forces its surrounding neighbours into the active state via hydrogen diffusion, and thereby into synchronisation. Whether two neighbouring facets synchronise is thus determined by whether the diffusional coupling strength is sufficient to overcome the difference in natural oscillation frequencies of neighbouring facets. The relative strength of coupling weakens with rising hydrogen pressure (see Supplementary Note 6) and at a certain threshold, desynchronization of the system occurs^43,44^.

## Discussion

Summarising, both experiments and model calculations show that catalytic hydrogen oxidation in a compartmentalised reaction nanosystem, consisting of differently oriented rhodium nanofacets, proceeds in a parallel, but not necessarily in a synchronised way. The system exhibits different reaction modes including a transition from self-sustaining oscillations to chaotic behaviour, as evidenced by correlation analysis and Fourier spectra of experimental data. The close agreement of the theoretical modelling, where chaos is unambiguously present, with the experimental observations provides additional evidence for the emergence of chaotic behaviour in the present reaction nanosystem.

The known transitions to chaos in chemical reactions, as observed on the meso- and macroscale, usually occur via temporal changes, such as period-doubling^38,39,57,72^ or unstable periodic orbits^73^. In the present nanosystem, however, weakening of coupling leads to emergence of chaos via spatial separation into several individually oscillating clusters.

Another peculiarity of the compartmentalised nanosystem is the role of diffusional coupling, which is much greater in nanosystems than in meso- and macroscale systems, due to the relation of the reactants diffusion length to the system size. In combination with fluctuations, which also become more important in small systems, this significantly complicates the observation of a low-dimensional chaotic behaviour. This could be overcome by a particular layout of the sample surface, consisting of nm-sized rhodium facets of different crystallographic orientations, and thorough adjustment of reaction conditions to vary the coupling strength in a range necessary to trigger transitions from synchronised to unsynchronised oscillations and eventually to chaos.

The reaction studied in the present work is relatively simple and the model system is well-defined. This allowed for model simulations using a system of 52 coupled oscillators, mimicking the used compartmentalised sample well. The absence of apparative noise and of reaction-induced fluctuations in model simulations, as well as the possibility of unlimited timeseries, provided an undisturbed insight into the origin of transitions between monofrequential and multifrequential self-sustaining oscillations, and chaotic behaviour. This makes limits of the experimental approach visible and points out a way for future studies: improving the time-resolution would allow for discrimination of coupling-induced and fluctuation-induced effects and may even allow in situ monitoring of the local hydrogen diffusion via surface density fluctuations^70^. The present study is a first attempt to transfer the existing comprehensive knowledge about chaos, collected for complex dynamical systems, to catalysis on a nanoscale. Demonstrating that variation of the coupling strength between the different nanofacets of a single nanoparticle can trigger transitions from synchronised behaviour to desynchronized oscillations and to chaos provides a deeper insight into the complexity of a heterogenous catalytic reaction. Accordingly, one can envision analogous future studies of more realistic catalysts and more complex reactions. Using the proper observation techniques, the possibility of nanoscale confinement and chaotic behaviour in small, compartmentalised chemical or even biological systems can be explored, building on the experimental and modelling routes of the present work.

## Methods

### Experimental setup

The experiments were conducted in an all-metal FIM/FEM setup, equipped with a specimen holder that can be operated at temperatures from 77 to 900 K, a channel-plate/screen assembly for imaging with noble gas ions, e.g., Ne^+^ (in the FIM mode) or by electrons (in the FEM mode), and a high purity gas supply system (O_2_: 99.999% Messer, H_2_: 99.97% Linde).

### Sample preparation

The Rh nanocrystal was fabricated by electrochemical etching of a Rh wire (0.1 mm, Mateck, 99.99%), followed by shaping the apex and cleaning the apex surface by field evaporation under UHV conditions at 77 K. The progress of shaping by field evaporation was controlled in situ by FIM.

### FEM/FIM measurements

The UHV chamber of the FIM/FEM setup was operated as a flow-reactor for catalytic hydrogen oxidation reaction. The required constant gas phase composition in the 10^−6^ to 10^−5^ mbar range during the FEM monitoring of the reaction was controlled by precision leak valves and verified by a residual gas analyser (QMS: MKS e-Vision 2). A recently developed automatised temperature control system^74^ was applied for high precision temperature control in the temperature range from 413 to 493 K. The Ni/CrNi thermocouple directly spot-welded to the shaft of the Rh nanocrystal served as a temperature sensor. FIM images during the sample preparation and the FEM images during the ongoing hydrogen oxidation were recorded by a CCD camera (Hamamatsu C13440).

### Construction of frequency maps

The recorded FEM video files were analysed by a fully automated algorithm that evaluates the local FEM intensity timeseries. Oscillating and non-oscillating timeseries were distinguished via the analysis of the autocorrelation-function and the local oscillation frequencies were determined by Fourier analysis. Details on the developed algorithm are given in the Supplementary Note 2.

### Calculation of the correlation matrix and clustering

The spatial correlations between the local FEM intensities extracted from the FEM video files recorded in situ were determined by computing the cross-correlation function. The correlation matrix was constructed from the maxima of the pairwise cross-correlation functions of individual superpixels. Using an agglomerative hierarchical clustering algorithm^75,76^, highly correlated superpixels were organised into clusters, which were displayed as colour-coded correlation maps. Additional information on the correlation clustering is given in Supplementary Note 4.

### Micro-kinetic model simulations

Micro-kinetic model simulations were carried out on the basis of the Langmuir-Hinshelwood mechanism of the hydrogen oxidation reaction and the formation/depletion of subsurface oxygen as the feedback mechanism governing the self-sustained oscillations^20,24,25,37^. Individual nanofacets on the surface of the Rh nanocrystal were modelled by a grid of 52 oscillators coupled by hydrogen diffusion considered by a corresponding term in the model simulations. Details regarding the micro-kinetic simulations are presented in Supplementary Note 6.

### Calculation of the maximum Lyapunov exponents

The maximum Lyapunov exponents were calculated using a standard numerical method^77^ via parallel computing utilising 100 threads on an Intel® Xeon® Platinum 8176 M CPU. The details of the procedure are given in Supplementary Note 8.

## Supplementary information

Supplementary Information

Description of Additional Supplementary Files

Supplementary Movie 1

Supplementary Movie 2

Supplementary Movie 3

Supplementary Movie 4

Supplementary Movie 5

Supplementary Movie 6

## Data Availability

The FEM video data and the results of the microkinetic modelling have been deposited in a Zenodo repository under 10.5281/zenodo.5973929. The parameters used for modelling are provided in the Supplementary Information.

## Code availability

All the code or mathematical algorithm files within this paper are available from the corresponding authors upon reasonable request.
