# Supplementary material for: Emergence of chaos in a compartmentalized catalytic reaction nanosystem
Source: Nat Commun. 2023 Feb 10;14:736. doi: 10.1038/s41467-023-36434-y (PMC9911747; doi:10.1038/s41467-023-36434-y)
Supplement: Supplementary file 1 — Supplementary Information [file 41467_2023_36434_MOESM1_ESM.pdf]

Emergence of chaos in a compartmentalized catalytic reaction nanosystem

Maximilian Raab[1], Johannes Zeininger[1], Yuri Suchorski[1], Keita Tokuda[2], Günther Rupprechter[1]*

[1]Institute of Materials Chemistry, TU Wien; Getreidemarkt 9, 1060 Vienna, Austria

[2]Department of Computer Science, University of Tsukuba; 1-1-1 Tennodai, Tsukuba, Ibaraki 305-8577, Japan

Supplementary Information

Supplementary Note 1: Characterization of the Rh nanocrystal

Both the FIM and FEM microscopes are point projection techniques[1,2]. An FIM projection image magnifies the hemispherical apex of a nanosized sample by a factor of~10^6 in a kind of stereographic projection (Supplementary Fig. 1a) and shows the positions of protruding surface atoms with atomic resolution, thus reflecting the crystallography of the imaged surface (Supplementary Fig. 1b).

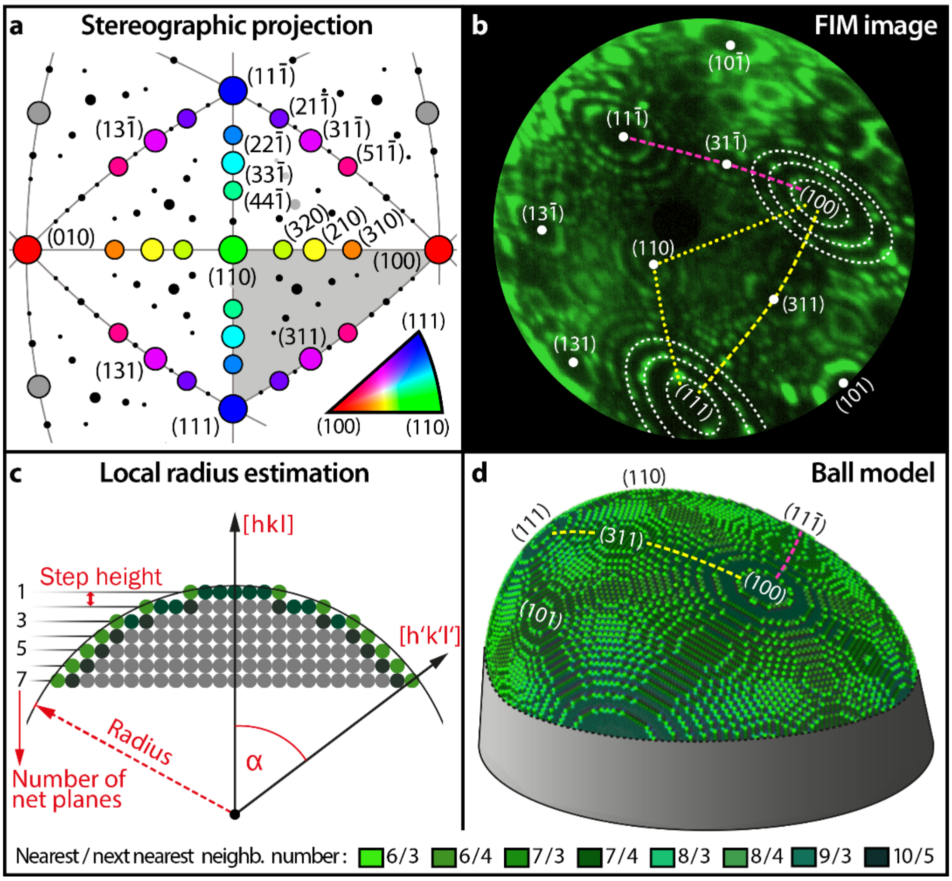

Supplementary Fig. 1. Crystallographic layout, size and shape of the Rh nanocrystal: (**a**) stereographic projection of a [110]-oriented hemispherical apex of an fcc-crystal. The conventional inverse pole figure (IPF) is shown in the bottom right corner. The gray-colored triangle in the projection corresponds to the IPF; (**b**) FIM image of the Rh nanocrystal obtained at T = 77 K using Ne^+ ions. Orientations of several facets and the top three circular atomic steps on the (100) and (111) facets are indicated. The purple and yellow dashed lines exemplarily mark the crystallographic zone lines between the $(11\bar{1})$, (100) and (111) facets. The yellow dotted lines complete the contour of the triangle, coloured grey in (a); (**c**) schematic drawing illustrating the “ring counting” method to estimate local radii of the apex. Circles represent atoms and are color-coded according to their nearest and next-nearest neighbour numbers (colour-code at the bottom); (**d**) ball model of the used nanocrystal apex with facets indicated, using the same colour-code as (c). The same zone lines are marked as in (b).

The atomically resolved FIM images are well-suited to characterize the crystallographic layout, size and shape of the imaged sample. Symmetry arguments, e.g., can be used to assign the crystallographic orientation to each visible nanofacet. In the present case of the Rh nanocrystal (Supplementary Fig. 1b), the centre facet is [110]-oriented, as visible from the stereographic

projection in Supplementary Fig. 1a. The remaining low-Miller-index facets are $\{111\}$ and $\{100\}$ facets (cf. Supplementary Fig. 1a and 1b). The radius of a crystalline hemispherical apex of a field emitter tip can be estimated by counting the number of atomic steps represented by net-rings in an FIM image between two poles of known angular separation[2]. Supplementary Fig. 1c shows that the number of net planes n multiplied by the step height provides the height of the apex cap determined by an angle α, so the tip apex radius r is given by

$$r = \frac{ns}{1 - \cos\alpha} \tag{1.1}$$

For the fcc-lattice, the step height of a (hkl) pole facet is provided by

$$s = \frac{a}{\delta\,\sqrt{h^2k^2l^2}}\,, \tag{1.2}$$

where a is the lattice constant (Rh: $a\ = 0.380\,\mathrm{nm}$) and δ is defined as 1 if h, k and l are all odd and 2 otherwise. This ring counting method allows determining the local tip radii from FIM micrographs and can, *e.g.*, be applied for Supplementary Fig. 1b along the $\{100\}$-$\{311\}$-$\{111\}$ line segments (highlighted by dashed lines). Due to the ellipsoid like shape of the tip apex, zone lines, that are supposed to be of the same length for a hemispherical tip apex, differ and provide differing local radii, i.e., 12 nm from the (100)-$(31\bar{1})$-$(11\bar{1})$ line (purple dashed line in Supplementary Fig. 1b) and 25 nm from the (100)-(311)-(111) line (yellow dashed line in Supplementary Fig. 1b). The FIM image basically visualizes solely protruding surface atoms, therefore it is useful to construct a ball model containing all surface atoms to obtain a full crystallographic understanding of the apex orientation, size and shape. To match the asymmetry in the projection image, the typical hemispherical approach in ball model construction was extended to an ellipsoidal approach, characterized by three ellipsoid parameters a, b and c instead of only one radius r. The best fit with the FIM images was achieved by an ellipsoid-like apex surface with shifted vertical axis, i.e., with horizontal axes of different lengths (a and a' in Supplementary Fig. 1a), using the model description proposed by Narushin et al.[3].

Supplementary Note 2: Construction of frequency maps

The frequency maps presented in Fig. 2 of the main text were constructed by digital analysis of the FEM video files recorded in situ during catalytic H_2 oxidation (Supplementary Movies 1-3). The automated algorithm evaluates local timeseries extracted from the FEM recording by separating oscillating from non-oscillating behaviour via autocorrelation-function analysis and, if possible, determining the frequency by Fourier analysis. The procedure behind the construction of a frequency map is visualized in Supplementary Fig. 2.

Supplementary Fig. 2a schematically shows a set of the recorded video frames, each consisting of 480 x 480 pixels corresponding to a field of view of 27 x 27 nm. To reduce the statistical noise and the calculation time, binning into superpixels, each consisting of 4 x 4 pixels, was carried out, resulting in a 120 x 120 grid of superpixels for the complete field of view. Then, for each superpixel, timeseries of the local evolution of the FEM intensity were extracted from the FEM videos, as exemplarily shown in Supplementary Fig. 2b for two superpixels *X* and *Y*.

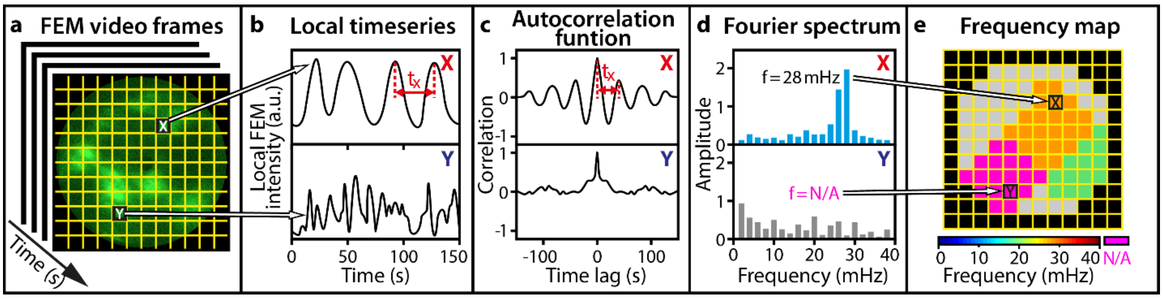

Supplementary Fig. 2. Construction of a frequency map: (**a**) a set of the recorded FEM video frames (field of view 27 by 27 nm, corresponding to 480 x 480 pixels or 120 x 120 superpixels); the X and Y indicate two exemplary superpixels; (**b**) local timeseries extracted from the superpixels X and Y shown in (a); (**c**) corresponding autocorrelation functions, the course of the function in lower panel indicates aperiodic behaviour; (**d**) Fast-Fourier transformation spectra calculated from the autocorrelation function shown in (c); in the oscillating case, the most prominent peak represents the frequency; (**e**) the calculated oscillation frequencies for superpixel timeseries can be displayed as a color-coded frequency map.

For oscillating systems, the oscillation frequency can be determined by Fourier analysis, which converts the temporal signal to the frequency domain. However, for superpixels with lower intensities, the determination of the main frequency by the automated algorithm can be impeded by noise and reaction-induced fluctuations superimposed on the oscillation timeseries. In order to increase the robustness of the algorithm in such cases, the autocorrelation function $\mathrm{ACF}(\tau)$ defined as

$$\mathrm{ACF}(\tau) = \int_{-\infty}^{+\infty} f(t)^* \cdot f(t+\tau) dt, \qquad \textbf{(2.1)}$$

where $f(t)$ is the timeseries, * denotes the complex conjugate and τ is the time lag, was first calculated from the local timeseries. For finite discrete functions, as the timeseries extracted from our FEM video data, the local $ACF(\tau)$ for the pixel X becomes

$$\mathrm{ACF}(\tau) = \langle I_X(t) I_X(t+\tau) \rangle_t, \qquad \textbf{(2.2)}$$

where $I_X(t)$ is the normalized measured FEM intensity of the superpixel X, and $\langle\ \rangle$ denotes the expectation value, i.e., the calculated $\mathrm{ACF}(\tau)$ measures the correlation of a timeseries with a lagged copy of itself.

In the case of a regular periodic oscillations, the $\mathrm{ACF}(\tau)$ displays a decaying, sine shaped progression with τ, with the same time difference between peaks as the original timeseries, as schematically shown in the upper row in Supplementary Fig. 2c. This inherent property allows determination of the oscillation frequency from the prominent peak of the Fourier spectrum (Supplementary Fig. 2d) resulting from the Fourier transformation of the $\mathrm{ACF}(\tau)$. This allows a reliable determination of the oscillation frequency also in the presence of noise and fluctuations of timeseries[4]. This method was applied in the frequency map algorithm (Supplementary Fig. 2).

In contrary, aperiodic behaviour results in the absence of pronounced periodic peaks and in a fast decay in the amplitude of the autocorrelation function[5], as schematically shown in the lower panel in Supplementary Fig. 2c. Therefore, the $\mathrm{ACF}(\tau)$ can be used also as indicator of

aperiodic behaviour (for details see Supplementary Note 3 and Supplementary Fig. 3). For a steady catalytic state, only reaction- or diffusion-induced fluctuations of the superpixel intensity are present[6] with the measured timeseries being similar to white noise, where the

$$\mathrm{ACF}(\tau) = \begin{cases} 1; & \tau = 0 \\ 0; & \tau > 0 \end{cases} \qquad \textbf{(2.3)}$$

To finally obtain the frequency map, the resulting main frequency values are plotted into a grid, using a defined colour-code (Supplementary Fig. 2e). Superpixels showing signs of aperiodic behaviour (f=N/A) are coloured pink in the frequency map (Supplementary Fig. 2e), while those in a steady state, i.e., with low intensity variations are coloured grey. The resulting map visualizes differences in the local frequencies of oscillating regions and discriminates regions with steady states or aperiodic behaviour that show indications of chaotic behaviour, discussed in Supplementary Note 3. The known positions of particular $\mathrm{Rh}(hkl)$ nanofacets (Supplementary Fig. 1) allow linking the gained information to the crystallographic layout of the nanocrystal surface.

Supplementary Note 3: Experimental indications of chaos

A reliable and frequently used indicator of chaotic behaviour in a dynamic system is an exponential growth of separation of infinitesimally close trajectories characterized by the Lyapunov exponents[7]. To calculate Lyapunov exponents for a particular system, exact knowledge of the temporal progression of the system variables and the consequent dimensionality of the system in phase space are required. While this prerequisite can be fulfilled for model calculations, experimental measurements in complex systems usually provide access to one or few observables $u(t)$ describing the system, with the system dimensionality often remaining unknown. The delay embedding theorem of Takens[8] provides a way to reconstruct the complete system behaviour from a sequence of a single variable $u(t)$, promising, in principle, a solution to the treatment of experimental signals.

However, to use this approach, near-infinite data sets and absence of noise are necessary. Both conditions are typically not given for experimental measurements due to finite length of measured timeseries and external noise introduced by the fluctuation of external parameters (e.g. temperature and partial pressures of reactants) as well as internal noise inherent to surface diffusion and reaction induced fluctuations[6]. This predicament makes the use of this approach hardly practicable: the number of data points N_{min} required to reliably reconstruct the complete system behaviour lies in the range of up to $N_{min} = 42^D$, where D is the upper bound of the system dimension[9]. This is hardly realizable in an experiment (130 million data points are necessary for $D=5$, which equals 30 days of continuous measurements at a rate of 50 datapoints per second. In nanoscale catalytic experiments the reaction conditions cannot be kept constant for such a long time). To summarize, the above arguments strictly limit the applicability of Takens theorem for reconstructing the system behaviour[10] and in turn also restrict the applicability of Lyapunov exponent calculations for experimental data[11].

Therefore, alternative chaos indicators should be considered for finite and noisy timeseries usually measured in an experiment to establish the initial assumption of chaotic behaviour. Recently, Carruba et al. used the autocorrelation function index as a chaos indicator to identify chaotic motion in celestial mechanics[12]. This autocorrelation function index (ACFI) is defined as

$$\text{ACFI} = \frac{1}{\tau_2 - \tau_1} \sum_{\tau=\tau_1}^{\tau_2} n(\tau), \qquad \textbf{(3.1)}$$

where τ_1 and τ_2 are the limits of the investigated time lag interval, and $n(\tau)$ is one if $|\text{ACF}(\tau)| > \Omega$ and zero otherwise, with Ω being a chosen threshold value. Regular states are associated with high values of ACFI, while chaotic states present substantially lower values. The choice of the parameters Ω, τ_1 and τ_2 does affect the values of ACFI slightly, however, does not change the relationship between ACFI for regular and chaotic states. A separation into regular and chaotic states is made by comparing the ACFI values of all investigated timeseries. This method was used, e.g., to identify chaotic behaviour caused by mean-motion dynamics of asteroid families in the solar system, as has been demonstrated e.g. on Veritas asteroid family, trans Neptunian objects and was also applied to the benchmark Hénon-Heiles system[12].
Supplementary Fig. 3a presents the autocorrelation function and ACFI values calculated for the timeseries measured for the $(31\bar{1})$ ROI at 453 K for the three different hydrogen pressures as shown in Fig. 2a-c of the main text. The substantial difference in the ACFI values between the periodic and aperiodic states (0.71 and 0.79 versus 0.09) is a strong indication for chaotic behaviour. In addition, the shape of the $ACF(\tau)$ curve can serve as an indicator: strong dampening of the ACF(τ) is characteristic for chaotic behaviour. Sivakumar et al. investigated the existence of chaos in rainfall-runoff processes using the exponential decay of the ACF(τ) as indication of chaotic behaviour[13]. The oscillating velocity of airflow through a cylindrical shell was found to be chaotic by means of the dampening of the ACF(τ) among other chaos indicators[14].

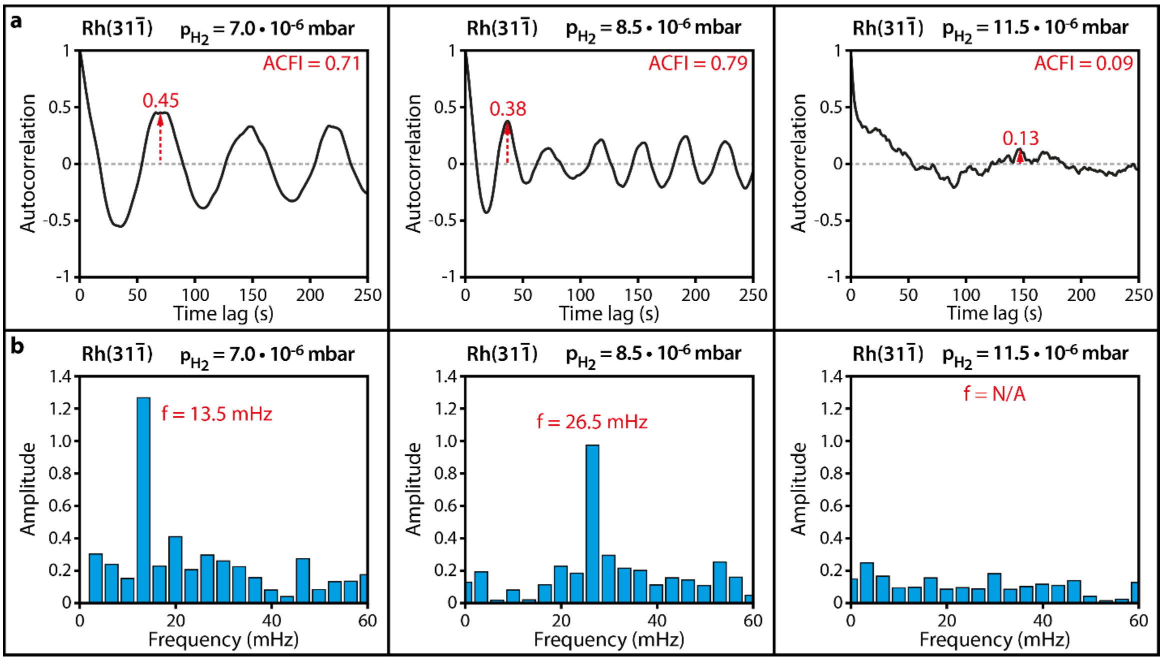

Supplementary Fig. 3. Chaos indicators calculated from experimental data: (**a**) autocorrelation functions calculated for the timeseries presented in Fig. 2a, b and c. The values of the second peak height of the autocorrelation function and of the respective calculated autocorrelation function indicator (ACFI) are shown; (**b**) Fourier spectra calculated from the same timeseries by means of fast-Fourier transformation with the frequency determined from the largest peak indicated.

In our experimental results, the heights of the second peak values of the $\mathrm{ACF}(\tau)$ show a clear difference between the regular (monofrequential: 0.45 and multifrequential: 0.38) and aperiodic (0.13) states (Supplementary Fig. 2a), which strongly hints at a chaotic state.
The Fourier spectra of measured timeseries showing broad band spectra without dominant peaks[15] can serve as alternative indicators of chaotic behaviour[16]. This idea was, e.g., utilized by Guzman et al. who used the shape of the Fourier spectra to follow the transition from laminar to chaotic flow in converging-diverging channels[17]. In the present study, Fourier spectra for the same timeseries (Fig. 2a-c of the main text) were calculated using the fast-Fourier transformation (see supplementary text S2 and Fig. S2) and are shown in Supplementary Fig. 3b. Regular oscillations shown in Fig. 2a and b provide prominent peaks from which the characteristic oscillation frequencies (13.5 and 26.5 mHz, correspondingly) can be determined, while the apparently aperiodic timeseries (Fig. 2c) results in the rather continuous spectrum. A prominent peak at higher frequencies is not present for $p_{H2} = 11.5 \times 10^{-6}$ mbar, the broad low-frequency band between 0 and 10 mHz results from accumulation of aperiodic noise.
Since chaotic systems generally exhibit a substantial drop in spatial correlation of ongoing processes[18–20], it is reasonable to prove the decay of spatial correlation of local instabilities in the present observations.

Supplementary Note 4: Calculation of the correlation matrix and correlative clustering
The FEM videos presented in this work show spatio-temporal patterns induced on the surface of a Rh nanocrystal by reaction/diffusion processes during catalytic hydrogen oxidation. Apart from the characterization of each local timeseries independently for their oscillation frequency and chaos indicators (see Supplementary Notes 2 and 3), determining the degree of spatial coupling between the reaction at different locations on the nanocrystal surface gives further indications of chaotic behaviour of the investigated system, as chaotic systems generally exhibit a substantial drop in spatial correlation[18–20]. To investigate the studied reaction/diffusion system for these additional indications of chaos necessitates the analysis of the spatial correlation between recorded timeseries of different crystallographic facets, an idea which has previously been successfully used to investigate fluctuations in catalytic CO oxidation on Pt nanotips[21,22].
To reveal regions with coherent oscillations, the spatial correlation between individual superpixels can be calculated and consecutively used to display regions of high mutual correlation as "correlation maps". An automated algorithm determines the spatial correlation between superpixels of the FEM recording by computing the cross-correlation function, followed by organizing highly correlated superpixels into clusters. The algorithm is based on the construction of a dendrogram from the individual correlation coefficients and the optimal number of clusters determined via optimization of the cluster quality. The resulting clusters of superpixels can then be visualized as color-coded correlation maps. The computational steps of this algorithm are depicted in Supplementary Fig. 4.
To perform the correlation analysis, original FEM video-frames taken in situ during catalytic H_2 oxidation on the Rh nanocrystal with a spatial resolution of 480 x 480 pixels were converted into a 60 x 60 grid of superpixels (8 x 8 pixels each) (Supplementary Fig. 4a). The local intensity evolution $f_i(t)$ of each superpixel is extracted from the original FEM video data as timeseries, as is shown in Supplementary Fig. 4b. Then, for each pair of individual superpixels, the cross-correlation function $\rho_{i \leftrightarrow j}(\tau, d)$ is calculated.

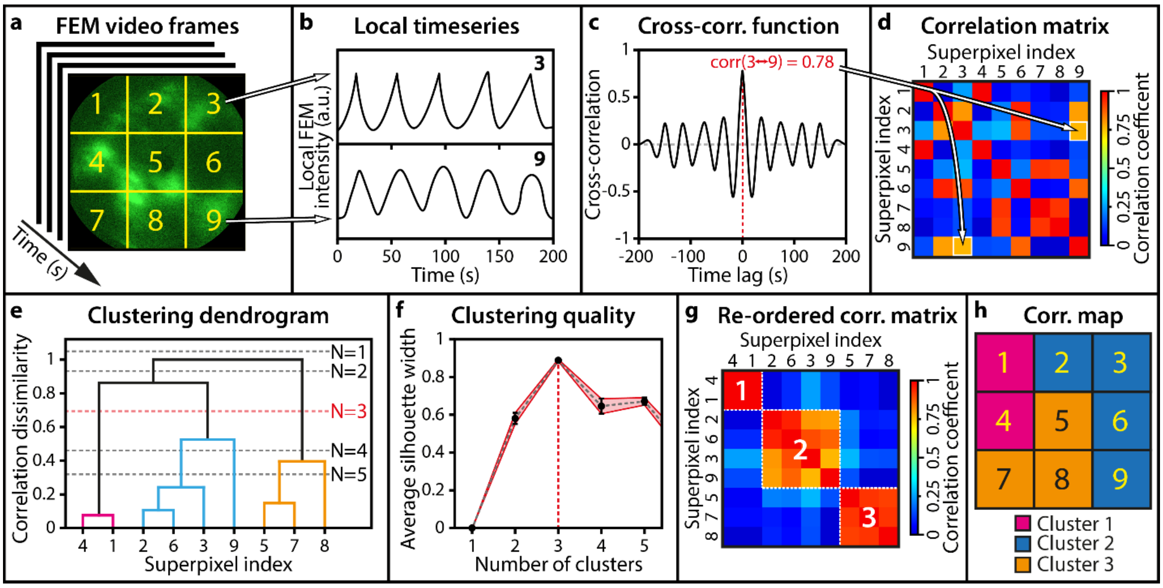

Supplementary Fig. 4. Calculation of the correlation matrix and correlation map: (**a**) recorded FEM video frames converted in the superpixel grid (here only 9 for simplicity) serve as spatio-temporal input signal; (**b**) local timeseries read out for two exemplary superpixels 3 and 9 from (a); (**c**) cross-correlation function calculated for the superpixels 3 and 9. The maximum value provides the spatial correlation coefficient, 0.78 in the present case; (**d**) correlation matrix constructed from the cross-correlation functions calculated for each pair of superpixels; (**e**) clustering dendrogram constructed by agglomerative hierarchical clustering based on the correlation matrix in (d), where different numbers of clusters can be chosen depending on the dissimilarity cut-off (dashed lines); (**f**) clustering quality measured as the average silhouette width over the entire correlation matrix for different numbers of clusters; (**g**) correlation matrix from (d), but re-ordered according to the clustering dendrogram in (e); (**h**) resulting correlation map, where each superpixel is coloured according to its affiliation to a respective cluster.

The cross-correlation function is defined as

$$\rho_{\mathrm{i}\leftrightarrow\mathrm{j}}(\tau, d) = \int_{-\infty}^{+\infty} f_i(t)^* \cdot f_j(t+\tau)dt, \qquad \textbf{(4.1)}$$

and contains information about the spatial correlation between the individual superpixels i and j separated by the distance d.

Here * denotes the complex conjugate and τ is the time lag as described in Supplementary Note 2. For finite discrete functions, as the timeseries extracted from our FEM video data, the cross-correlation function becomes

$$\rho_{\mathrm{i}\leftrightarrow\mathrm{j}}(\tau, d) = \langle I_i(t) I_j(t+\tau)\rangle_t, \qquad \textbf{(4.2)}$$

where $I_i(t)$ and $I_j(t)$ are the normalized measured intensities on the superpixels i and j, respectively, and $\langle\,\rangle$ denotes the expectation value. The maximum values of the cross-correlation functions are considered as the spatial correlation coefficients $\rho_{i\leftrightarrow j}(\tau_{max}, d)$. The resulting values of the spatial correlation coefficients can then be summarized in a correlation matrix, which can be visualized as a color-coded grid, where the colour of each field represents the absolute spatial correlation coefficient between two superpixels (Supplementary Fig. 4d).

An analogous procedure can also be applied to calculate the correlation matrix for the micro-kinetic modelling (Fig. 4c of the main text) by replacing the experimentally measured timeseries by the timeseries of calculated surface coverages on each oscillator.

Subsequently, the superpixels or oscillators are grouped into clusters with high mutual correlation (Supplementary Fig. 4e). This is achieved via applying an agglomerative hierarchical clustering algorithm[23,24]. The procedure starts with each object (i.e. superpixel or oscillator) being its own cluster, so that the number of clusters equals the number of objects. At each clustering step, the two most similar clusters are merged, leading to a new clustering with one less available cluster. This procedure is performed until all initial clusters are joined into one single cluster containing all objects. To start the procedure, i.e. to find the two most similar objects, the object dissimilarity value

$$d(\mathrm{i} \leftrightarrow \mathrm{j}) = 1 - \rho_{i \leftrightarrow j}(\tau_{\max}, d) \quad \textbf{(4.3)}$$

based on the spatial correlation coefficients $\rho_{i \leftrightarrow j}(\tau_{\max}, d)$ between the objects was used. The next clustering steps are performed using the maximal cluster dissimilarity value

$$D(a, b) = \max_{x_a \in C_a, x_b \in C_b} d(x_a, x_b), \quad \textbf{(4.4)}$$

where a and b are the merged clusters and $d(x_a, x_b)$ are the dissimilarities of the clusters a and b to all other clusters.

The resulting hierarchy can be visualized as a tree, called dendrogram, which illustrates how the clusters are merged from the lowest to the highest level and at which value of dissimilarity D the linkage between each pair of clusters happens (Supplementary Fig. 4e). By cutting the dendrogram at particular levels of dissimilarity, different partitions into final numbers of clusters N can be obtained. This can be done by either choosing a distance criterion (defined cut-off level) or a number criterion (defined number of final clusters). In this work, we used a modified number criterion, first determining which final number of clusters would yield the best clustering by calculating the clustering quality in the form of the average silhouette width[25] over all clustered objects for all possible cluster numbers (Supplementary Fig. 4f).

As a measure of the clustering quality, the silhouette $s(i)$ introduced by Rousseeuw[26] as

$$s(i) = \frac{b(i) - a(i)}{\max\limits_{i \in A}\{a(i), b(i)\}} \text{ with } b(i) = \min_{C \neq A} d(i, C), \quad \textbf{(4.5)}$$

was used, where $a(i)$ is the average dissimilarity of i to all other objects of the cluster A containing i and $d(i, C)$ is the average dissimilarity of i to all objects in any cluster C different from A. The silhouette can be understood as a measure of how similar an object is to other objects in its own cluster when compared to objects in the next similar cluster. A high silhouette value indicates high clustering quality, meaning that the respective object is well matched to its own cluster and ill-matched to all other clusters. In order to rate the quality of the whole data set, one considers the overall average silhouette width, which is simply the average over all $s(i)$. Calculating this value for all different sets of clusters resulting from the dendrogram allows choosing the appropriate cut-off in the dendrogram by selecting the cluster number which maximizes the average silhouette. Such procedures are often used to choose the optimal number of clusters, e.g., in fuzzy clustering[27] or k-means clustering[28].

In the next step, the correlation matrix (Supplementary Fig. 4d) is reordered according to the clustering and seriated using the DendSer algorithm[29], i.e., ordered in a way that maximizes the correlation between adjacent rows/columns (Supplementary Fig. 4g). Such reordering illustrates high intra-cluster correlations (red squares in the reordered correlation matrix, and low extra-cluster correlations represented by a blue surrounding region). Finally, since each superpixel from the videoframes (Supplementary Fig. 4a) necessarily belongs to some cluster, the superpixels belonging to the same cluster with high correlation can be marked by the same colour and presented as a correlation map (Supplementary Fig. 4h). The areas of the same colour in Supplementary Fig. 4g illustrate surface areas with strong mutual correlation, e.g., pixels 1 and 4 (purple colour) correspond to the surface area with highest correlation of ongoing reaction process, since they belong to the cluster 1 in Supplementary Fig. 4g. An analogous procedure can be applied to the microkinetic calculations in exactly the same way as for the experiments, with the superpixel timeseries replaced by the calculated surface coverages for each respective oscillator.

We applied the above-described algorithm to calculate the spatial correlation matrices and construct correlation maps for the experimental timeseries exemplarily shown in Fig. 2 of the main text. The results show a high spatial global synchronization for the monofrequential oscillations in Fig. 2a represented by a single cluster in the correlation map in Fig. 3b (left hand side) for the oscillating region. The multifrequential oscillations also exhibit a strong spatial correlation within the zones of the same frequency, as is visible from the comparison of frequency map in Fig. 2b with correlation map in Fig. 3b (middle). The aperiodic mode (Fig. 2c) yields only weakly correlated regions, as is apparent from several smaller clusters formed in the correlation map in Fig. 3b (right hand side). This low degree of spatial correlation is a property indicating the possibility of spatio-temporal chaos[19].

Supplementary Note 5: Fluctuations in the active steady state

In the active steady state of catalytic hydrogen oxidation on Rh, the active facets of the nanotip exhibit a low coverage of both hydrogen and oxygen. The high mobility of hydrogen causes its density fluctuations, reminding of those of CO molecules in catalytic CO oxidation[30]. Such diffusion-induced fluctuations are desynchronized even within individual facets and have a monomodal Gauss-like amplitude distribution similar to that of white noise[30].

Supplementary Fig. 5 shows the analysis of the fluctuations in the catalytically active steady state of hydrogen oxidation on the investigated nanotip at 453 K and at constant $p_{H2} = 13.0 \times 10^{-6}$ mbar and $p_{O2} = 4.4 \times 10^{-6}$ mbar (exemplary videoframe in Fig. 5a). The FEM intensity read out from the same ROIs as in Fig. 2 produces noisy timeseries (Supplementary Fig. 5b). Applying the algorithm explained in Supplementary Note 4, the spatial correlation matrix for the active steady state was calculated (Supplementary Fig. 5c). As characteristic for diffusion induced fluctuations, only a negligible spatial correlation between different superpixels was found.

The striking difference between the correlation matrix in Fig 3a (right panel), attributed to the chaotic behaviour, and the correlation matrix in Supplementary Fig. 5c, reflecting the fluctuations, allows a distinction between these two different processes.

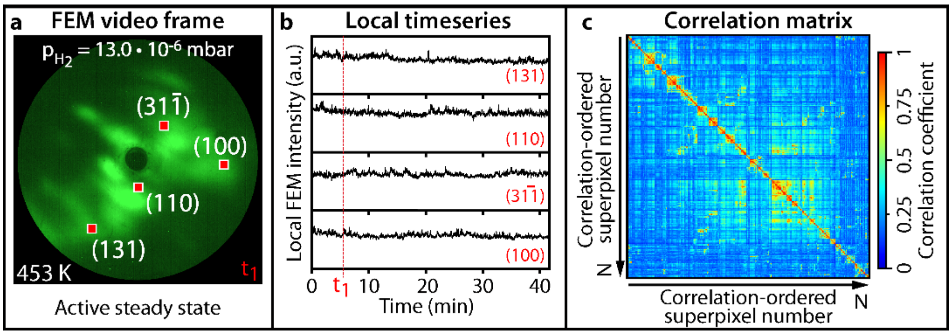

Supplementary Fig. 5. Fluctuations in the active steady state at constant $p_{H2} = 13.0 \times 10^{-6}$ mbar, $p_{O2} = 4.4 \times 10^{-6}$ mbar and T = 453 K; (**a**) in situ FEM video frame with ROIs placed in the (110), (100), (131) and (31$\bar{1}$) regions; (**b**) local FEM intensity curves measured within the above ROIs. The time point corresponding to the video frame in (a) is marked as t_1; (**c**) correlation coefficient matrix summarizing the correlation between experimental timeseries of each individual super pixel (8 x 8 pixels), the colour code represents the value of the correlation coefficient.

Supplementary Note 6: Micro-kinetic model simulations

Since, in contrary to experimental data, calculated timeseries are not subject to the data point number limitations, it is reasonable to use microkinetic model simulations for the rationalization of observed aperiodic behaviour. The used model is based on the Langmuir-Hinshelwood mechanism and on the periodic formation and depletion of subsurface oxygen as the feedback mechanism governing the oscillations. Both mechanisms are well established for oscillating hydrogen oxidation on Rh[31–35] and are explained in Supplementary Fig. 6.

Oxygen and hydrogen adsorb competitively on the Rh surface. Oxygen has a much higher initial sticking coefficient and preferentially adsorbs on the surface via a molecular precursor (Supplementary Fig. 6 R1) that then dissociates to two chemisorbed oxygen atoms (Supplementary Fig. 6 R2). In the oxygen covered, catalytically inactive state, adsorbed oxygen atoms migrate to subsurface positions, forming subsurface oxygen (Supplementary Fig. 6 R3). Presence of subsurface oxygen reduces the sticking coefficient of oxygen, eventually leading to a favoured adsorption of hydrogen, which also occurs dissociatively (Supplementary Fig. 6 R4). The shifted equilibrium in competitive adsorption switches the system to its active state, where only low coverages of both hydrogen and oxygen are present due to the continuous formation of water (Supplementary Fig. 6 R5, OH intermediates are not considered in the model), which immediately desorbs from the surface under the given reaction conditions[36,37]. In the active state, the subsurface oxygen is migrating back to the surface and is consumed in the reaction (Supplementary Fig. 6 R3). Due to the depletion of the subsurface oxygen depot, the sticking coefficient of oxygen increases again. Consequently, oxygen from the gas phase again adsorbs preferentially, and the reaction returns to the initial inactive state, closing the oscillation cycle[31–35]. The above mechanism can therefore be described by five reaction equations (Supplementary Fig. 6 R1 to R5).

This model concept was originally introduced by McEwen et al. to simulate field-modified oscillations observed by FIM[38] and contained terms describing the reduction of the activation barrier for oxygen incorporation by a high electric field of > 10 V/nm[39,40]. Since in the present case of an FEM study field-effects can be neglected (see Supplementary Note 7), in contrast to

the FIM studies by McEwen et al., the field-dependent terms were omitted in the present modelling.

In the present work, a novel element, namely coupling between different nanofacets, was introduced. In a catalytic reaction, such coupling, which plays an important role in the reaction accompanying spatio-temporal processes, can occur via gas phase[41] or via surface diffusion of reactants[34,42]. In the present low-pressure conditions, coupling occurs via diffusion of hydrogen atoms between adjacent nanofacets[38], while the oxygen diffusion, which is very slow under the present conditions, can be neglected[39,40].

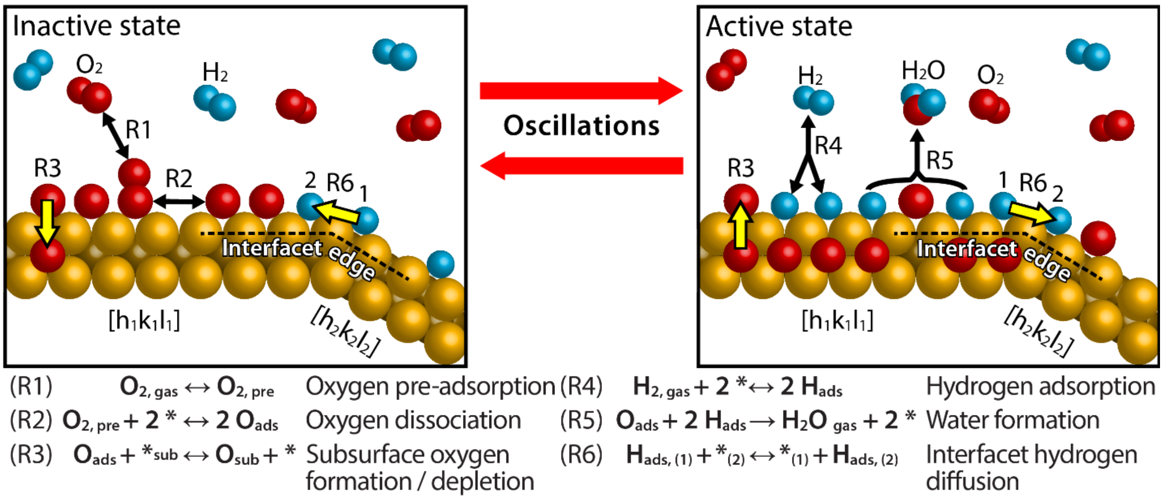

Supplementary Fig. 6. Schematic illustration of the mechanism of self-sustaining oscillations in catalytic hydrogen oxidation on Rh nanofacets. Colour-code: Rh (golden), O (red), and H (blue). In the corresponding reaction equations R1 – R6 the (*) and ($*_{\text{sub}}$) represent empty surface and subsurface sites, respectively. The index (1) or (2) describes the surface sites of a hydrogen atom diffusing across interfacet edges.

Since our experimental system consists of many differently oriented nanofacets able to oscillate independently, it is reasonable to consider a system of coupled oscillators as an appropriate model. The corresponding model network is based on the crystallographic layout of the present nanocrystal surface (see Fig. 1, Supplementary Note 1 and Supplementary Fig. 1), with individual oscillators representing nanofacets as schematically shown in Fig. 4a. The diffusive coupling between adjacent oscillators (Supplementary Fig. 6 R6) is incorporated into the model by addition of a corresponding diffusion term. In our extended model, the hydrogen coverage θ_H^i, the oxygen coverage θ_O^i and the subsurface coverage θ_S^i are described by the following kinetic equations:

$$\frac{\mathrm{d}\theta_H^i}{\mathrm{d}t} = 2k_\mathrm{a}^\mathrm{H} p_{\mathrm{H}_2} {\theta_*^i}^2 - 2k_\mathrm{d}^\mathrm{H} {\theta_H^i}^2 - 2k_\mathrm{r}^i \theta_H^i \theta_O^i + k_\mathrm{dif}^\mathrm{H} \sum_{j\neq i}^{n} C_{i,j}\left(\theta_H^j \theta_*^i - \theta_H^i \theta_*^j\right), \tag{6.1}$$

$$\frac{\mathrm{d}\theta_O^i}{\mathrm{d}t} = \frac{2}{1 + K^i {\theta_*^i}^2}\left(k_\mathrm{a}^\mathrm{O} K^i p_{\mathrm{O}_2} {\theta_*^i}^2 - k_\mathrm{d}^{\mathrm{O},i} {\theta_O^i}^2\right) - k_\mathrm{ox}\theta_O^i\left(1 - \theta_S^i\right) + k_\mathrm{red}^i \theta_S^i \theta_*^i - k_\mathrm{r}^i \theta_H^i \theta_O^i \tag{6.2}$$

$$\frac{\mathrm{d}\theta_S^i}{\mathrm{d}t} = k_\mathrm{ox}\theta_O^i\left(1 - \theta_S^i\right) - k_\mathrm{red}^i \theta_S^i \theta_*^i \tag{6.3}$$

where i is the index of the respective oscillator, and θ_*^i represents the empty surface sites defined as $\theta_*^i = 1 - \theta_H^i - \theta_O^i$. The rate and coupling constants are given by

$$k_{\mathrm{a}}^{\mathrm{H}} = S_0^{\mathrm{H}}\, a_{\mathrm{s}} / \sqrt{2\pi m_{\mathrm{H}_2}/\beta} \quad \textbf{(6.4)}$$

$$k_{\mathrm{d}}^{\mathrm{H}} = k_{\mathrm{d0}}^{\mathrm{H}} e^{-\beta E_{\mathrm{d}}^{\mathrm{H}}} \quad \textbf{(6.5)}$$

$$k_{\mathrm{r}}^{i} = k_{\mathrm{r}}^{0} e^{-\beta\left(E_{\mathrm{r}} + A_{\mathrm{r}}^{\mathrm{H}}\theta_H^i + A_{\mathrm{r}}^{\mathrm{O}}\theta_O^i\right)} \quad \textbf{(6.6)}$$

$$k_{\mathrm{dif}}^{\mathrm{H}} = k_{\mathrm{dif0}}^{\mathrm{H}} e^{-\beta E_{\mathrm{dif}}^{\mathrm{H}}} \quad \textbf{(6.7)}$$

$$C_{i,j} = w_{i,j}/{d_{i,j}}^2 \quad \textbf{(6.8)}$$

$$K^{i} = K_0 e^{-\beta\left(E_{\mathrm{K}} + A_{\mathrm{K}}^{\mathrm{O}}\theta_O^i + A_{\mathrm{K}}^{\mathrm{S}}\theta_S^i\right)} \quad \textbf{(6.9)}$$

$$k_{\mathrm{a}}^{\mathrm{O}} = S_0^{\mathrm{O}}\, a_{\mathrm{s}} / \sqrt{2\pi m_{\mathrm{O}_2} k_{\mathrm{B}} T} \quad \textbf{(6.10)}$$

$$k_{\mathrm{d}}^{\mathrm{O},i} = k_{\mathrm{d0}}^{\mathrm{O}} e^{-\beta\left(E_{\mathrm{d}}^{\mathrm{O}} + A_{\mathrm{d}}^{\mathrm{O}}\theta_O^i + B_{\mathrm{d}}^{\mathrm{O}}{\theta_O^i}^2\right)} \quad \textbf{(6.11)}$$

$$k_{\mathrm{ox}} = k_{\mathrm{ox}}^{0} e^{-\beta E_{ox}^{i}} \quad \textbf{(6.12)}$$

$$k_{\mathrm{red}}^{i} = k_{\mathrm{red}}^{0} e^{-\beta\left(E_{\mathrm{red}}^{i} + A_{\mathrm{red}}^{\mathrm{S}}\theta_S^i\right)}, \quad \textbf{(6.13)}$$

where S_0^H corresponds to the initial sticking coefficient of hydrogen, a_s denotes the area of a surface site and is set to 11 Å^2, m_{H_2} is the average molecular mass of hydrogen and $\beta = 1/k_{\mathrm{B}}\,T$. The parameters S_0^{O} and m_{O_2} represent the initial sticking coefficient of oxygen and its molecular mass, respectively.

The present model was extended by the parameters $C_{i,j}$, which corresponds to the distance-weighted coupling coefficient between oscillators i and j, and $d_{i,j}$, the distance between the respective oscillators. The $w_{i,j}$ values are coupling factors, generally equal to 1 (marked by black double arrows in Fig. 4a) apart from oscillators coupled through the reconstruction-induced diffusion barrier located at central (110) zone. For such barrier-reduced coupling, marked by blue double arrows in Fig. 4a, a coupling factor of 0.2 was assumed. Most of the kinetic parameters in the present calculations were used for calculations of oscillation frequencies on a curved Rh microcrystal[32] and are summarized in the Supplementary Table 1 below. The stability of the calculated results to small changes of kinetic parameters was ensured by routinely performed extensive testing.

Supplementary Table 1. Parameters used in the micro-kinetic model simulations of the coupled oscillator network. The activation energy values for subsurface oxygen formation and reduction are given in the text. Energies are given in eV, rate constants in 1/s and k_{dif0}^{h} in nm²/s.

symbol	parameter description	value
S_0^H	initial sticking coefficient of H	0.232
k_{d0}^h	pre-factor for hydrogen desorption	3.0×10^{10}
E_d^H	desorption energy of H	0.64
k_r^0	pre-factor for water formation	7.0×10^{12}
E_r	activation energy for water formation	0.79
A_r^H	coverage dependence of activation energy of water formation on H	-0.27
A_r^O	coverage dependence of activation energy of water formation on O	-0.145
k_{dif0}^h	pre-factor for hydrogen diffusion	4.5×10^{2}
E_{dif}^H	activation energy for hydrogen diffusion	0.187
K_0	pre-factor for oxygen dissociation equilibrium constant	0.2525
E_k	activation energy for oxygen dissociation equilibrium constant	-0.178
A_K^O	coverage dependence of adsorbed oxygen on oxygen dissociation	0.158
A_K^s	coverage dependence of sub-surface oxygen on oxygen dissociation	0.075
S_0^O	initial sticking coefficient of O	0.95
k_{d0}^O	pre-factor for oxygen desorption	6.0×10^{13}
E_d^O	desorption energy of O	3.2
A_d^O	coverage dependence of oxygen desorption energy on adsorbed oxygen	-0.5
B_d^O	coverage dependence of oxygen desorption energy on molecular oxygen	-0.7
k_{ox}^0	pre-factor for oxygen diffusion from surface to sub-surface sites	2.0×10^{12}
k_{red}^0	pre-factor for oxygen diffusion from sub-surface to surface sites	1.85×10^{13}
A_{red}^s	coverage dependence of sub-surface oxygen on surface subsurface reduction	0.27

The natural oscillation frequency of each facet, i.e., without coupling to other regions, is largely determined by its rate of subsurface oxygen formation/depletion, which acts as feedback mechanism. This rate depends on the local activation energy for the formation of subsurface

oxygen (E_{ox}^i in the model equations)[31–33], which is related to the crystallographic structure of the Rh surface, i.e. its local atomic surface roughness[43]. Facets with higher density of steps and kinks have been shown to oxidize faster and consequently possess lower E_{ox}^i values. Accordingly, depending on the crystallographic facet it represents, every oscillator of the model system was assigned an E_{ox}^i value in the range from 1.09 to 1.36 eV (Fig. 4a), similar to the range, chosen in our recent study of nano-pacemakers[34].

The present ellipsoid-like nanocrystal exhibits unequal a axes (Fig. 1), i.e., the two halves of the ellipsoid exhibit a different curvature, which also leads to a different number of steps and kinks on the nanofacets of the same orientations located on different halves. The facets on the upper half (Fig. 4a), represented by oscillators 1-26, are more stepped and kinked and therefore atomically rougher, which is considered in the model by lowering their respective E_{ox}^i values in comparison with the equivalent facets at the bottom half (oscillators 27-52 in Fig. 4a).

The activation energy E_{red}^i for subsurface oxygen reduction can be calculated from the respective E_{ox}^i values using the relation[31,]

$$E_{\text{red}}^i = 0.293 + 0.776\, E_{\text{ox}}^i. \qquad \textbf{(6.14)}$$

The values of E_{ox}^i for each type of facet and the diffusion coupling factor $w_{i,j}$ across the central barrier were refined until the observed oscillation phenomena occurred in the simulations.

Chaotic behaviour of systems can reliably be identified by its sensitive dependence on the initial conditions via the exponential growth of the distance between the trajectories starting infinitesimally close to each other in phase space[7]. This property of a chaotic of system can be characterized by expensive numerical methods, i.e., calculation of the maximal Lyapunov exponents, as explained in detail in Supplementary Note 8. However, it is more feasible and descriptive to additionally investigate the dependence of the simulated system behaviour on small perturbations of the initial conditions. For this purpose, we modified the initial conditions (θ_H^i, θ_O^i, θ_S^i of each oscillator) by adding a small perturbation to the coverages of each oscillator and repeated the calculation with otherwise the same parameter set. The perturbation vector added to the initial condition was randomly drawn from a $3n$-dimensional multivariate normal distribution with a mean of 0, and the Euclidian norm of the perturbation was normalized to 10^{-6} before adding.

An exemplary result of such perturbation is shown in Fig. 4b, where the upper stripes in each of three panels show the unperturbed timeseries, the perturbed ones are shown in the middle stripes and the differences between these two timeseries are shown in the bottom stripes. Since the small change in the initial conditions has no influence on the long-term behaviour of a periodic process, a perpetual difference value of zero for all oscillators is a characteristic sign of process periodicity. This is indeed the case in the two upper panels of Fig. 4b. For chaotic systems, a small perturbation in the initial conditions is initially also hardly observable in the difference plot. However, with progressing time, the initially small perturbations of the system grow very quickly, leading to an evidently aperiodic progression of the timeseries and to a positive maximum Lyapunov exponent. This behaviour can be clearly observed for our simulations at $p_{H2} = 9.0 \times 10^{-6}$ mbar, strongly indicating a deterministic chaotic behaviour (lower panel in Fig. 4b). Increasing the hydrogen pressure reduces both the spatial synchronization and the temporal periodicity of the system, leading to multifrequential oscillations and then to a chaotic state. The reduction of spatial correlation in the simulations is reflected in the

correlation coefficient matrices of Fig. 4c, showing small highly correlated regions and a low correlation between different regions. Such an effect was also observed experimentally at higher hydrogen pressures (Fig. 3a), as evident by the same particular appearance of the respective correlation coefficient matrices. This similarity in the appearance of the experimentally determined and calculated correlation coefficient matrices provides a strong additional evidence of chaotic behaviour of our reaction system.

The loss of spatial and temporal synchronization can be explained as follows: due to differing pressure dependencies of the hydrogen adsorption rate (governing the natural frequency) and diffusion rate (governing the coupling), the relation between the coupling synchronizing the oscillations and the trend to maintain natural oscillation frequencies of individual facets changes. The relative weakening of the coupling strength leads to the desynchronization of the system[44,45]. This results in the loss in spatial correlation and a transition from monofrequential to multifrequential oscillations and ultimately to chaos.

Supplementary Note 7: The role of the electric field

As mentioned above, the field emission microscope (FEM) is a projection microscope in which the surface of the apex of a tip-shaped nm-sized sample is imaged by electrons field emitted via their tunnelling into vacuum due to an applied electrostatic field of 3 to 5 V/nm[1]. Therefore, the possibility of field effects on the studied phenomena, in the present case the catalytic H_2 oxidation reaction, should be considered. The kinetics of catalytic H_2 oxidation is governed by the energetics of interactions of hydrogen and oxygen with the catalyst surface. Thus, a possible field effect must have its origin in the dependence of the binding energies of O and H atoms on the electrostatic field close to the substrate surface. The sole possible reason for such a dependence is the field induced redistribution of electron density near the metal surface, influencing the binding of chemisorbed species. Such a redistribution was the subject of previous experimental and theoretical studies, most of which are recapitulated in a review[46]. The main message of these studies is that a high electrostatic field applied to a metal surface leads to a measurable modification of the surface electron density, which depends on the applied field strength, metal and the distance to the surface, as illustrated in Supplementary Fig. 7[47].

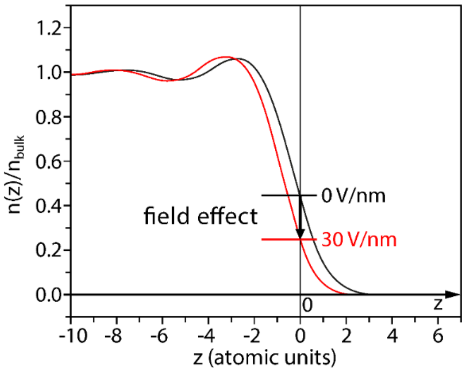

Supplementary Fig. 7. Field-free and field-modified electron density near the metal surface n(z) characterized by the Wigner-Seitz radius $r_s = 3.0$ a.u. (1 a.u. = 0.529 Å) as function of the distance z from the surface. The black line corresponds to zero field, the red line to an externally applied field of 30 V/nm directed away from the surface. Note the direction of the applied electrostatic field: away from the surface (positively charged surface). A similar electron

density modification by a field directed into the surface (negatively charged surface) is prohibited by the onset of field emission. Based on Ref. 47.

Such a field induced redistribution of surface electron density may lead to an observable modification of the binding energy of adsorbates, as directly measured on an atomic scale using a sophisticated microspectroscopy technique (field ion appearance energy spectroscopy, FIAES) for CO[48–50], O_2[50,51], N_2[52] and other adsorbates, as summarized in review[53]. The field-induced modifications of the binding energy have indeed an impact on the reaction kinetics, as directly measured for CO oxidation on Pt[54].

However, the above measurements and calculations show that observable changes in the binding energy of adsorbates take place only at applied field strengths significantly above ~10 V/nm. This sets physical limits for a possible field-effect, since only a "positive" field of such strength, i.e., a field directed away from the surface (positively charged sample surface) as, e.g., in a field ion microscope (FIM), can be applied to a metal surface. Such a field "pushes" electrons into the bulk (red curve in Supplementary Fig. 7). Any attempt to apply a "negative" electric field of field strengths necessary for modification of binding energy of adsorbates to a metal surface leads to the avalanche like field emission, resulting eventually in explosive electron emission[55]and destruction of the surface. Therefore, field effects on the binding energy of adsorbates such as oxygen, hydrogen, or carbon monoxide can not arise in an FEM, since the sample in an FEM is always negatively charged and thus the field strengths necessary for a field effect can not be reached, due to the physical limits described above. Consequently, the absence of a field-effect on the binding energy of adsorbates excludes field effects on the kinetics of CO and H_2 oxidation in a FEM, as previously discussed in detail[54,56]. For CO oxidation, this has been directly confirmed experimentally by using a pulsed voltage supply with varying duty pulses[54].

To ultimately ensure the absence of electric field effects in the present FEM studies of H_2 oxidation, additional pulsed-field experiments were performed (Supplementary Fig. 8) in analogy to earlier experiments on CO oxidation[54]. During these measurements, the field necessary for the FEM imaging resulted, instead from a constant voltage (Supplementary Fig. 8a), from a pulsed high-voltage supply with the same magnitude, but with a short pulse duration (Supplementary Fig. 8b). This way, a field free time up to 99% of the total measurement time could be achieved. Since the frequency of self-sustained oscillations is very sensitive to the binding energy of adsorbates (which would vary if the applied field would change the electron density distribution near the surface), the frequency of oscillations was taken into focus. The experiments showed no influence of the applied field used for FEM imaging. The direct comparison of the Fourier spectra for monofrequential self-sustaining oscillations at $p_{O2} = 4.4 \times 10^{-6}$ mbar, $p_{H2} = 5.0 \times 10^{-6}$ mbar and 453 K, monitored using the constant or pulsed field supply (Supplementary Fig. 8c) illustrates this convincingly. In addition, no differences between the results obtained using both modes of FEM imaging were observed for kinetic transitions between the active and inactive steady states. It also has to be noted that field strengths applied in FEM (e.g., 3.4 V/nm in the present experiments) are, in contrary to those applied in FIM, comparable and often even less than those used in scanning tunnelling microscopy (STM), where bias voltages from a few tenths to one volt, or several volts, are applied at Ångstrom distances.

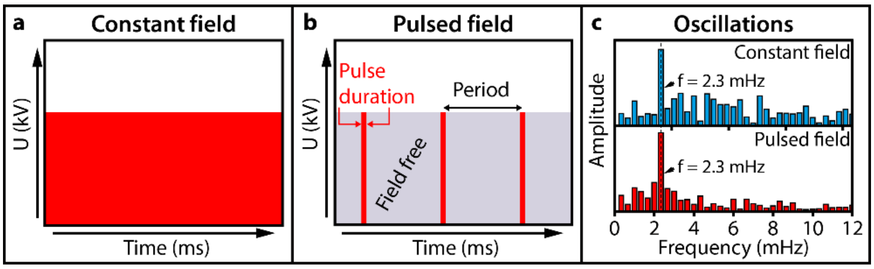

Supplementary Fig. 8. Constant field versus pulsed field supply in FEM imaging of H_2 oxidation on Rh: **a**) schematics of the constant voltage supply (constant applied field); (**b**) the same for the pulsed voltage supply. Pulses with duration of 200 µs and a period of 15 ms between the pulses (repetition frequency of 66.6 Hz) result in 99% of the total measurement time as field-free; (**c**) Fourier spectra of self-sustained oscillations in H_2 oxidation at p_{O2} = 4.4 x 10^{-6} mbar, p_{H2} = 5.0 x 10^{-6} mbar and 453 K with constant and pulsed applied imaging field.

Supplementary Note 8: Calculation of the maximum Lyapunov exponents

The calculation of the maximum Lyapunov exponents has been performed with the standard numerical method, i.e., taking the temporal average of the local expansion rate along a trajectory[57–59]. Because of the complexity of the Jacobian matrix of the current model, the original system of differential equations was integrated directly with a small perturbation to the trajectory instead of using the variational equation to calculate the local expansion rate. The procedure can be explained as follows: First, a short time interval T and a small value d used as the norm of the perturbation from the original trajectory are chosen. The values d and T should be chosen sufficiently small to provide that the evolution of the trajectory perturbation within the time interval T can be regarded as linear, but d should be sufficiently larger than the accuracy of the numerical integration. The initial perturbation vector $\delta x(t_0)$ was drawn from a multivariate normal distribution along with the choice of an initial condition, $x_0(t_0) = \{\theta_H^i(t_0), \theta_O^i(t_0), \theta_S^i(t_0)\}_i \in \mathbb{R}^{3n}$ in the phase space. The Euclidian norm of $\delta x(t_0)$ was normalized to $\|\delta x(t_0)\| = d$.

Then, a simultaneous integration of the differential equations for both initial conditions, $x_0(t_0)$ and $x_1^0(t_0) = x_0(t_0) + \delta x(t_0)$, is performed for the time interval $[t_0\ t_0 + T]$ to obtain the estimates of the two trajectories at $t_0 + T$, $x_0(t_0 + T)$ and $x_1^0(t_0 + T)$. The estimated local expansion is

$$\alpha_0 = \frac{x_1^0(t_0 + T) - x_0(t_0 + T)}{d} \tag{8.1}$$

Then, the next perturbation vector is calculated by normalizing the difference between two trajectories to the value d by

$$\delta x(t_0 + T) = d \cdot \frac{x_1^0(t_0 + T) - x_0(t_0 + T)}{\|x_1^0(t_0 + T) - x_0(t_0 + T)\|} \tag{8.2}$$

Then, for the initial conditions, $x_0(t_0 + T)$ and $x_1^1(t_0 + T) = x_0(t_0 + T) + \delta x(t_0 + T)$, and for the time interval $[t_0 + T\ t_0 + 2T]$, integration is performed again to obtain the local expansion at the next step

$$\alpha_1 = \frac{x_1^1(t_0 + 2T) - x_0(t_0 + 2T)}{d} \tag{8.3}$$

Likewise, successive integrations of a perturbed trajectory and normalization of the perturbation yield successive local expansions as

$$\alpha_k = \frac{x_1^k(t_0 + (k+1)T) - x_0(t_0 + (k+1)T)}{d} \quad \textbf{(8.4)}$$

The maximum Lyapunov exponent can be estimated by the following average with a sufficiently large value of L:

$$h_L = \frac{1}{LT}\sum_{k=0}^{L} ln\, \alpha_k. \quad \textbf{(8.5)}$$

The parameter values used in the current study are $d = 10^{-5}, T = 50s, L = 2000$. For calculating the pressure dependency of the maximum Lyapunov exponent (Fig. 4d), the same values of the initial conditions $x(t_0)$ were used for all parameter sets. This allowed to avoid the variability of the estimated exponent values arising from convergences to different attractors. The calculation of maximum Lyapunov exponent values shown in Fig. 4d required an equivalent of 9.3 years of the model time (number of different values along the p_{H2} parameter axis $\times\, T \times L \times 2 = 61 \times 50s \times 2000 \times 2 = 12200000s$).

Supplementary References

1. Good, R. H. & Müller, E. W. Field Emission. in *Electron-Emission Gas Discharges I / Elektronen-Emission Gasentladungen I* vol. 4 / 21 176–231 (Springer Berlin Heidelberg, 1956).
2. Müller, E. W. & Tsong, T. T. *Field ion microscopy: principles and applications*. (American Elsevier Pub. Co, 1969).
3. Narushin, V. G., Romanov, M. N. & Griffin, D. K. Egg and math: introducing a universal formula for egg shape. *Annals NY Academy of Science* **1505**, 169–177 (2021).
4. Sudhakar, R., Agarwal, R. & Roy, S. D. Frequency estimation based on iterated autocorrelation function. *IEEE Trans. Acoust., Speech, Signal Process.* **33**, 70–76 (1985).
5. Anishchenko, V. S., Vadivasova, T. E., Okrokvertskhov, G. A. & Strelkova, G. I. Correlation Analysis of Deterministic and Noisy Chaos. **48**, 11 (2003).
6. Suchorski, Yu., Beben, J., James, E. W., Evans, J. W. & Imbihl, R. Fluctuation-Induced Transitions in a Bistable Surface Reaction: Catalytic CO Oxidation on a Pt Field Emitter Tip. *Phys. Rev. Lett.* **82**, 1907–1910 (1999).
7. Cencini, M., Cecconi, F. & Vulpiani, A. *Chaos: from simple models to complex systems*. (World Scientific, 2010).
8. Takens, F. Detecting strange attractors in turbulence. in *Dynamical Systems and Turbulence, Warwick 1980* (eds. Rand, D. & Young, L.-S.) vol. 898 366–381 (Springer Berlin Heidelberg, 1981).
9. Smith, L. A. Intrinsic limits on dimension calculations. *Physics Letters A* **133**, 283–288 (1988).
10. Casdagli, M., Eubank, S., Farmer, J. D. & Gibson, J. State space reconstruction in the presence of noise. *Physica D: Nonlinear Phenomena* **51**, 52–98 (1991).

11. Ruelle, D. The Claude Bernard Lecture, 1989 - Deterministic chaos: the science and the fiction. *Proc. R. Soc. Lond. A* **427**, 241–248 (1990).

12. Carruba, V., Aljbaae, S., Domingos, R. C., Huaman, M. & Barletta, W. Chaos identification through the autocorrelation function indicator (ACFI). *Celest Mech Dyn Astr* **133**, 38 (2021).

13. Sivakumar, B., Berndtsson, R., Olsson, J. & Jinno, K. Evidence of chaos in the rainfall-runoff process. *Hydrological Sciences Journal* **46**, 131–145 (2001).

14. Gholami, I., Amabili, M. & Païdoussis, M. P. Dynamic divergence of circular cylindrical shells conveying airflow. *Mechanical Systems and Signal Processing* **166**, 108496 (2022).

15. Voorsluijs, V. & De Decker, Y. Emergence of chaos in a spatially confined reactive system. *Physica D: Nonlinear Phenomena* **335**, 1–9 (2016).

16. Herbst, B. M. & Ablowitz, M. J. Numerically induced chaos in the nonlinear Schrödinger equation. *Phys. Rev. Lett.* **62**, 2065–2068 (1989).

17. Guzmán, A. M. & Amon, C. H. Transition to chaos in converging–diverging channel flows: Ruelle–Takens–Newhouse scenario. *Physics of Fluids* **6**, 1994–2002 (1994).

18. Tufillaro, N. B., Ramshankar, R. & Gollub, J. P. Order-Disorder Transition in Capillary Ripples. *Phys. Rev. Lett.* **62**, 422–425 (1989).

19. Cross, M. C. & Hohenberg, P. C. Pattern formation outside of equilibrium. *Rev. Mod. Phys.* **65**, 851–1112 (1993).

20. Nakagawa, N. & Kuramoto, Y. Collective Chaos in a Population of Globally Coupled Oscillators. *Progress of Theoretical Physics* **89**, 313–323 (1993).

21. Suchorski, Yu., Beben, J. & Imbihl, R. Fluctuations during catalytic CO oxidation on different crystal planes of a Pt field emitter. *Progress in Surface Science* **59**, 343–354 (1998).

22. Suchorski, Yu. *et al.* Fluctuations and critical phenomena in catalytic CO oxidation on nanoscale Pt facets. *Phys. Rev. B* **63**, 165417 (2001).

23. Murtagh, F. & Contreras, P. Algorithms for hierarchical clustering: an overview. *WIREs Data Mining Knowl Discov* **2**, 86–97 (2012).

24. *Handbook of Cluster Analysis*. (Chapman and Hall/CRC, 2015). doi:10.1201/b19706.

25. Kaufman, L. & Rousseeuw, P. J. *Finding groups in data: an introduction to cluster analysis*. (Wiley, 2005).

26. Rousseeuw, P. J. Silhouettes: A graphical aid to the interpretation and validation of cluster analysis. *Journal of Computational and Applied Mathematics* **20**, 53–65 (1987).

27. Subbalakshmi, C., Krishna, G. R., Rao, S. K. M. & Rao, P. V. A Method to Find Optimum Number of Clusters Based on Fuzzy Silhouette on Dynamic Data Set. *Procedia Computer Science* **46**, 346–353 (2015).

28. Kodinariya, T. M. & Makwana, P. R. Review on determining number of Cluster in K-Means Clustering. *International Journal of Advance Research in Computer Science and Management Studies* **1**, 90–95 (2013).

29. Earle, D. & Hurley, C. B. Advances in Dendrogram Seriation for Application to Visualization. *Journal of Computational and Graphical Statistics* **24**, 1–25 (2015).

30. Suchorski, Y. Surface Diffusion Via Adsorbate Density Fluctuations. in *Encyclopedia of Interfacial Chemistry* 648–665 (Elsevier, 2018). doi:10.1016/B978-0-12-409547-2.12943-9.

31. Suchorski, Y. *et al.* Visualizing catalyst heterogeneity by a multifrequential oscillating reaction. *Nat Commun* **9**, 600 (2018).

32. Suchorski, Y. *et al.* Surface-Structure Libraries: Multifrequential Oscillations in Catalytic Hydrogen Oxidation on Rhodium. *J. Phys. Chem. C* **123**, 4217–4227 (2019).

33. Suchorski, Y. *et al.* Resolving multifrequential oscillations and nanoscale interfacet communication in single-particle catalysis. *Science* **372**, 1314–1318 (2021).

34. Zeininger, J. *et al.* Single-Particle Catalysis: Revealing Intraparticle Pacemakers in Catalytic H_2 Oxidation on Rh. *ACS Catal.* **11**, 10020–10027 (2021).

35. Winkler, P. *et al.* Coexisting multi-states in catalytic hydrogen oxidation on rhodium. *Nat Commun* **12**, 6517 (2021).

36. Gregoratti, L. *et al.* Structural effects on water formation from coadsorbed H + O on Rh(100). *Surface Science* **340**, 205–214 (1995).

37. Verheij, L. K. & Hugenschmidt, M. B. On the mechanism of the hydrogen–oxygen reaction on Pt(111). *Surface Science* **416**, 37–58 (1998).

38. McEwen, J.-S., Gaspard, P., Visart de Bocarmé, T. & Kruse, N. Nanometric chemical clocks. *Proc. Natl. Acad. Sci. U.S.A.* **106**, 3006–3010 (2009).

39. McEwen, J.-S., Gaspard, P., Visart de Bocarmé, T. & Kruse, N. Oscillations and Bistability in the Catalytic Formation of Water on Rhodium in High Electric Fields. *J. Phys. Chem. C* **113**, 17045–17058 (2009).

40. McEwen, J.-S., Gaspard, P., Visart de Bocarmé, T. & Kruse, N. Electric field induced oscillations in the catalytic water production on rhodium: A theoretical analysis. *Surface Science* **604**, 1353–1368 (2010).

41. Imbihl, R., Ladas, S. & Ertl, G. Spatial coupling of autonomous kinetic oscillations in the catalytic CO oxidation on Pt(110). *Surface Science* **215**, L307–L315 (1989).

42. Gorodetskii, V., Lauterbach, J., Rotermund, H.-H., Block, J. H. & Ertl, G. Coupling between adjacent crystal planes in heterogeneous catalysis by propagating reaction–diffusion waves. *Nature* **370**, 276–279 (1994).

43. Winkler, P. *et al.* How the anisotropy of surface oxide formation influences the transient activity of a surface reaction. *Nat Commun* **12**, 69 (2021).

44. Kuramoto, Y. Self-entrainment of a population of coupled non-linear oscillators. in *International Symposium on Mathematical Problems in Theoretical Physics* (ed. Araki, H.) vol. 39 420–422 (Springer-Verlag, 1975).

45. Kuramoto, Y. *Chemical Oscillations, Waves, and Turbulence.* (Springer International Publishing).

46. Suchorski, Yu., Schmidt, W. A., Ernst, N., Block, J. H. & Kreuzer, H. J. Electrostatic fields above individual atoms. *Progress in Surface Science* **48**, 121–134 (1995).

47. Kostrobiy, P. P., Markovych, B. M. & Suchorski, Y. Revisiting Local Electric Fields on Close-Packed Metal Surfaces: Theory Versus Experiments. *SSP* **128**, 219–224 (2007).

48. Schmidt, W. A., Suchorski, Yu., Block, J. H., Kreuzer, H. J. & Wang, R. L. C. Field ion appearance energy spectroscopy of CO^+ originated from Rh(111) and Au(111) surface step sites. *Surface Science* **326**, 243–251 (1995).

49. Suchorski, Yu., Schmidt, W. A., Block, J. H. & Medvedev, V. K. Appearance energy spectroscopy of CO^+ field ions emitted from W(111). *Surface Science* **331–333**, 277–282 (1995).

50. Sieben, B., Suchorski, Yu., Bozdech, G. & Ernst, N. Interaction of CO and O_2 with Pt Studied by Field Ion Appearance Energy Spectroscopy. *Zeitschrift für Physikalische Chemie* **202**, 103–115 (1997).

51. Suchorski, Y. Field ionization of O_2 on oxygen-covered Pt(111) and W(111) as studied using field ion appearance energy spectroscopy. *Ultramicroscopy* **73**, 139–145 (1998).

52. Suchorski, Yu., Medvedev, V. K. & Block, J. H. Noble-gas-like mechanism of localized field ionization of nitrogen as detected by field ion appearance energy spectroscopy. *Applied Surface Science* **94–95**, 217–223 (1996).

53. Suchorski, Y. Probing adsorption on a nanoscale: field desorption microspectroscopy. *Adsorption* **23**, 217–224 (2017).

54. Suchorski, Y., Imbihl, R. & Medvedev, V. K. Compatibility of field emitter studies of oscillating surface reactions with single crystal measurements: catalytic CO oxidation on Pt. *Surface Science* **401**, 392–399 (1998).

55. Litvinov, E. A., Mesyats, G. A. & Proskurovskiĭ, D. I. Field emission and explosive electron emission processes in vacuum discharges. *Sov. Phys. Usp.* **26**, 138–159 (1983).

56. Datler, M. *et al.* Hydrogen Oxidation on Stepped Rh Surfaces: µm-Scale versus Nanoscale. *Catal Lett* **146**, 1867–1874 (2016).

57. Shimada, I. & Nagashima, T. A Numerical Approach to Ergodic Problem of Dissipative Dynamical Systems. *Progress of Theoretical Physics* **61**, 1605–1616 (1979).

58. Benettin, G., Galgani, L., Giorgilli, A. & Strelcyn, J.-M. Lyapunov Characteristic Exponents for smooth dynamical systems and for hamiltonian systems; a method for computing all of them. Part 1: Theory. *Meccanica* **15**, 9–20 (1980).

59. Ott, E. *Chaos in dynamical systems*. (Cambridge University Press, 2002).
