# Supplementary material for: Emergence of chaos in a compartmentalized catalytic reaction nanosystem
Source: Nat Commun. 2023 Feb 10;14:736. doi: 10.1038/s41467-023-36434-y (PMC9911747; doi:10.1038/s41467-023-36434-y)
Supplement: Supplementary file 2 — Description of Additional Supplementary Files [file 41467_2023_36434_MOESM2_ESM.pdf]

Description of Additional Supplementary Files

File Name: Supplementary Movie 1
Description: *In situ* field emission microscopy (24 x speed) at p_{H2} = 7.0 x 10^{-6} mbar. Self-sustaining oscillations occurring during the hydrogen oxidation reaction on the ellipsoidal tip apex of a $[110]$-oriented rhodium nanocrystal consisting of nm-sized facets of different crystallographic orientation. The video shows monofrequential oscillations coupled on the whole tip surface, resulting from coupling *via* surface diffusion of hydrogen.

File Name: Supplementary Movie 2
Description: *In situ* field emission microscopy (24 x speed) at p_{H2} = 8.5 x 10^{-6} mbar. Multifrequential self-sustaining oscillations occurring during the hydrogen oxidation reaction on the ellipsoidal tip apex of a $[110]$-oriented rhodium nanocrystal consisting of nm-sized facets of different crystallographic orientation.

File Name: Supplementary Movie 3
Description: *In situ* field emission microscopy (24 x speed) at p_{H2} = 11.5 x 10^{-6} mbar. Spatio-temporal chaotic behaviour occurring during the hydrogen oxidation reaction on the ellipsoidal tip apex of a $[110]$-oriented rhodium nanocrystal consisting of nm-sized facets of different crystallographic orientation.

File Name: Supplementary Movie 4
Description: Microkinetic model simulations (24 x speed) at p_{H2} = 7.6 x 10^{-6} mbar. Calculated monofrequential oscillations of the simulated hydrogen coverages on 52 individual oscillators coupled *via* hydrogen diffusion.

File Name: Supplementary Movie 5
Description: Microkinetic model simulations (24 x speed) at p_{H2} = 8.3 x 10^{-6} mbar. Calculated multifrequential oscillations of the simulated hydrogen coverages on 52 individual oscillators coupled *via* hydrogen diffusion.

File Name: Supplementary Movie 6
Description: Microkinetic model simulations (24 x speed) at p_{H2} = 9.0 x 10^{-6} mbar. Calculated chaotic variations of the simulated hydrogen coverages resulting on 52 individual oscillators coupled *via* hydrogen diffusion.
